# Narrowband deep-blue emission from a BN-embedded cyclophane: synthesis, characterization, and OLED application

**DOI:** 10.1093/nsr/nwaf250

**Published:** 2025-07-03

**Authors:** Tianjiao Fan, Cheng Qu, Lian Duan, Yuewei Zhang

**Affiliations:** Key Lab of Organic Optoelectronics and Molecular Engineering of Ministry of Education, Department of Chemistry, Tsinghua University, Beijing 100084, China; Key Lab of Organic Optoelectronics and Molecular Engineering of Ministry of Education, Department of Chemistry, Tsinghua University, Beijing 100084, China; Key Lab of Organic Optoelectronics and Molecular Engineering of Ministry of Education, Department of Chemistry, Tsinghua University, Beijing 100084, China; Laboratory of Flexible Electronics Technology, Tsinghua University, Beijing 100084, China; Laboratory of Flexible Electronics Technology, Tsinghua University, Beijing 100084, China

**Keywords:** BN-embedded cyclophane, multi-resonance, narrowband, organic light-emitting diodes, deep-blue emission

## Abstract

Macrocyclic compounds featuring narrowband emission represent critical advancements for next-generation wide-color-gamut displays. In this work, we report the rational design and synthesis of an ultra-narrowband boron- and nitrogen-embedded cyclophane (BN-CP) through a one-pot triple intramolecular Bora-Friedel–Crafts reaction using aza[1_4_]cyclophane as the precursor. X-ray crystallography and density functional theory calculations highlighted the role of increased conformational rigidity after cyclization in facilitating spectral narrowing. Furthermore, BN-CP demonstrated narrowband deep-blue thermally activated delayed fluorescence, characterized by a full width at half maximum of 24 nm, arising from the synergistic multiple resonance effects of its three boron and nitrogen centers. The corresponding organic light-emitting diode achieved a peak external quantum efficiency of 23.3%, positioning it among the highest-performing deep-blue MR-OLEDs reported to date.

## Teaser

A novel BN-embedded cyclophane, synthesized in one-step, delivers ultra-narrowband deep-blue emission (full width at half maximum of 24 nm) and exceptional OLED performance (maximum external quantum efficiency of 23.3%) through rigid macrocyclic design.

## INTRODUCTION

**Figure 1.**
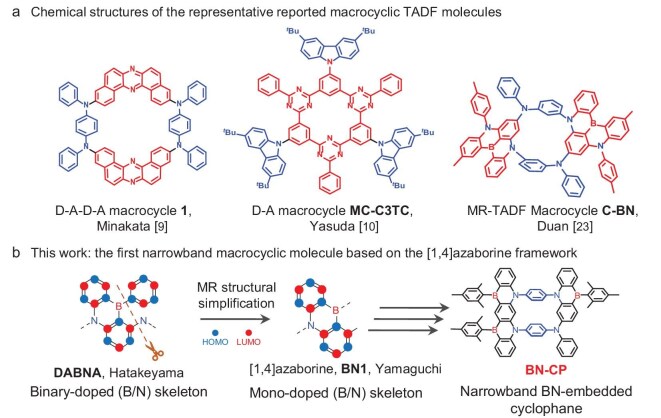
Macrocyclic TADF molecule design comparison. (a) Examples of macrocyclic TADF molecules and (b) our proposed BN-embedded cyclophane in this work.

The design and synthesis of novel functional macrocyclic molecules is a cornerstone of supramolecular chemistry research and has driven significant advancements in key technologies and scientific disciplines [1–3]. Recent developments in luminescent macrocyclic systems, particularly cyclic thermally activated delayed fluorescence (TADF) compounds, have become a focal point in materials science [4–10]. These TADF macrocyclic materials offer dual advantages: they efficiently utilize both singlet (*S*_1_) and triplet (*T*_1_) excitons for light emission and have constrained spatial configurations that suppress luminescent core aggregation and molecular motion. The implementation of this structural control has been demonstrated to enhance the performance of organic light-emitting diodes (OLEDs). For instance, Minakata *et al.*’s donor-acceptor-donor-acceptor (D-A-D-A) cyclic system achieved an external quantum efficiency (EQE) of 11.6%, surpassing the 6.9% EQE of its linear counterpart (Fig. 1a) [9]. Yasuda *et al.*’s parallel work demonstrated that the D-A type macrocycle MC-C3TC has a reduced S_1_-T_1_ energy gap (*ΔE*_ST_) and an improved photoluminescence quantum yield compared to the non-cyclic CTC molecule [10]. Ultimately, it achieved a 15.7% EQE in device applications.

While significant advances have been made in TADF macrocycles, critical challenges persist in spectral engineering. The broad emission spectra, resulting from vibronic coupling and excited-state structural relaxation, significantly impede their application in advanced displays necessitating a broad color gamut. Conventional spectral narrowing approaches through optical filters or microcavity architectures incur substantial energy loss, compromising device efficiency. Therefore, developing inherently narrowband macrocyclic TADF emitters is critical. Inspired by the multiple resonance TADF (MR-TADF) model, which involves alternating highest occupied molecular orbital (HOMO) and lowest unoccupied molecular orbital (LUMO) [11–22], we developed the first narrowband emissive macrocyclic compound. This unique boron (B)/nitrogen (N)-doped calix[4]arene derivative (C-BN) achieves >5.0 Å intermolecular spacing between MR-active cores, effectively suppressing aggregation-induced quenching and spectral broadening through steric confinement (Fig. 1a) [23]. OLEDs incorporating C-BN demonstrate ultra-pure blue electroluminescence (*λ*_EL_ = 452 nm) with a maximum external quantum efficiency (EQE_max_) of 26.6%.

To advance the molecular design of narrowband emitters, we developed a boron-/nitrogen-embedded cyclophane (BN-CP) through macrocyclization engineering. This [1,4]azaborine-derived system was synthesized via a one-pot triple intramolecular Bora-Friedel–Crafts reaction using aza[1_4_]cyclophane precursors (Fig. 1b). In contrast to previously documented diphenylamine-based [1,4]azaborines such as BN1 [24], L-BN [25], α-3BNOH [26], and α-3BNMes [27], the increased conformational rigidity upon post-cyclization enabled BN-CP to manifest an ultra-narrowband deep-blue emission with a peak at 430 nm, a small full width at half maximum (FWHM) of 24 nm, and Commission Internationale de l’Eclairage (CIE) coordinates of (0.17, 0.01). To our knowledge, BN-CP constitutes the first narrowband macrocyclic molecule based on the [1,4]azaborine framework, demonstrating the efficacy of macrocyclic design in spectral narrowing. Leveraging its high photoluminescence quantum yield (PLQY = 97.0%), the fabricated OLED achieved an EQE_max_ of 23.3%.

## RESULTS AND DISCUSSION

### Synthesis

**Figure 2.**
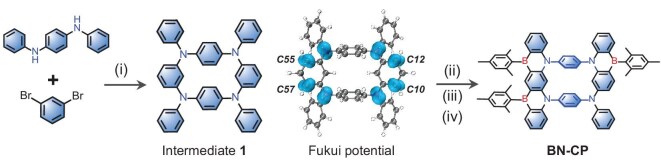
Synthesis of BN-CP reagents and conditions: (i) Pd_2_(dba)_3_, X-Phos, NaO(*t*-Bu), dry xylene, 140°C, 24 h. 61%; (ii) BBr3, o-dichlorobenzene, 185°C, 48 h; (iii) DIPEA, o-dichlorobenzene, 185°C, 72 h; (iv) MesMgBr, 45°C, 24 h, 14.7%.

The synthesis of BN-CP is depicted in Fig. 2 and detailed in the Supporting Information (Experimental Section). The macrocyclic precursor 1 was efficiently prepared via a one-step Buchwald–Hartwig coupling reaction using commercially available reagents. A one-step Bora-Friedel–Crafts-type reaction was successfully carried out based on the fact that the carbon atoms (C10, C12, C55, and C57) of intermediate 1 have the most negative dual descriptor potential, as determined by Fukui potential [28–30]. This involved sequential treatment with 12 equivalents of BBr_3_ (180°C, 48 h), 15 equivalents of diisopropylethylamine (180°C, 72 h), and 20 equivalents of bromo(2,4,6-trimethylphenyl)magnesium (MesMgBr, 45°C, 24 h), resulting in the target macrocyclic TADF compound BN-CP in a 14.7% yield. Furthermore, the steric hindrance provided by the mesityl groups effectively prevented both borylation at undesired reactive sites and excessive borylation. Consistent with conventional MR materials, BN-CP exhibited good solubility in commonly employed solvents (toluene, dichloromethane, and tetrahydrofuran). The chemical structures of all newly synthesized intermediates and final products were confirmed using NMR spectroscopy and high-resolution mass spectrometry (Figs S17–S22).

### X-ray single-crystal analysis

**Figure 3.**
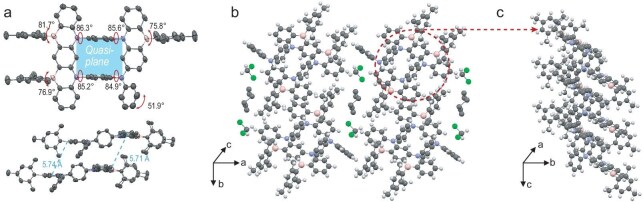
Single-crystal structures of BN-CP with 50% thermal ellipsoid probability. (a) Selected angles between the central bridging benzene ring and the MR fragments (upper), distance between two neighboring MR planes of BN-CP (below). (b, c) Packing of BN-CP along the c-axis to form porous columns.

The precise molecular architecture of BN-CP was elucidated by single crystal X-ray diffraction analysis (Fig. 3), obtained by gradual diffusion of n-hexane vapor into CH_2_Cl_2_ solutions. BN-CP crystallizes in a triclinic P1 space group consisting of 2 BN-CP, 2 n-hexane, and 2 CH_2_Cl_2_ molecules within a primitive unit cell (Table S1). As shown in Fig. S1, the B-C and N-C bond lengths in BN-CP range from 1.436 to 1.453 Å and 1.531 to 1.571 Å, respectively, consistent with the bond lengths characteristic of aromatic [1,4]azaborine frameworks (Fig. S1) [25,26,31]. The planar configuration of the [1,4]azaborine core imparts a quasi–two-dimensional rigid structure to the macrocyclic cavity of BN-CP. The angles between the central bridging benzene ring and the MR fragments on either side are ∼85°. Consequently, in contrast to conventional [1,4]azaborine structures with flexible benzene ends, the cyclized configuration significantly reduces the structural deformation from the ground state to the excited state as well as structural relaxation in the excited state, thereby enhancing spectral narrowing and increasing the PLQY. Within the crystal lattice, the macrocyclic units align along the c-axis, resulting in highly ordered assemblies that form porous columns with a cavity diameter of 7.43 Å (Fig. S2). The presence of nearly orthogonal benzene linkages and peripheral mesityl groups induces a slipped stacking arrangement of the BN-CP molecules within the columnar structure, without significant π-π interactions. A layered architecture emerges between the columns, interspersed with dichloromethane and n-hexane molecules along the a-axis. This unique arrangement not only contributes to the stability of the overall structure, but also plays a key role in the electronic properties of the material. Moreover, single crystals of intermediate 1 were also grown via slow solvent evaporation from a dichloromethane/petroleum ether mixture (Fig. S3). In contrast to the crystal structure of BN-CP (∼85°), the dihedral angles between the two benzene rings and the adjacent substituents were markedly smaller (∼48°), demonstrating that the intramolecular macrocyclic strain increases upon introduction of the boron moieties. Therefore, triple borylation might be a compromise of enhanced macrocyclic ring tension despite negative Fukui potentials favoring another form of borylation.

### Photophysical properties

**Figure 4.**
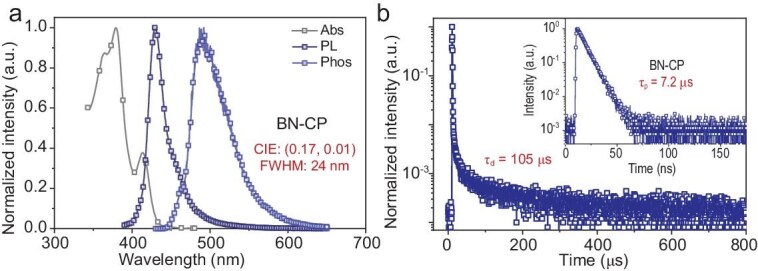
Photophysical characterizations. (a) UV-vis, fluorescence, and phosphorescence spectra of BN-CP in dilute toluene solution (10^−5^ M). (b) Transient PL spectrum of BN-CP in oxygen-free toluene solution at 298 K.

The photophysical properties of BN-CP were analyzed in dilute toluene solutions (1 × 10^−5^ M), and the results are presented in Fig. 4 and Table 1. The compound displays characteristic MR-TADF absorption with a distinct peak at 414 nm, which is indicative of short-range charge transfer (SR-CT) transitions. BN-CP demonstrates an ultra-narrowband deep-blue emission, peaking at 430 nm with a FWHM of 24 nm, corresponding to the CIE coordinates of (0.17, 0.01), which contrasts sharply with the broad spectra observed in traditional diphenylamine-based [1,4]azaborines like BN1, BN2, and L-BN (Figs S4 and S5) [24,25]. This validates our molecular design approach. The solvent effect of BN-CP was also investigated (Fig. S6). Upon transitioning from low-polarity cyclohexane to high-polarity dichloromethane, spectroscopic analysis revealed minimal bathochromic shifts—specifically, an 8 nm redshift in absorption and a 15 nm redshift in emission. These modest solvatochromic effects provide compelling evidence for the SR-CT character of the molecule's singlet state. Furthermore, due to the minimal vibrational coupling and negligible structural reorganization between the ground and excited states of the macrocyclic architecture, BN-CP achieved an exceptionally high PLQY of 97.0% through suppressed vibrational coupling and minimal ground/excited-state structural reorganization.

**Table 1.** The physical data of BN-CP.

Emitter	*λ*_abs_ ^a^ [nm]	*λ*_em_ ^a^ [nm]	FWHM^a^ [nm]	*E*_S1_ ^a^ [eV]	*E*_T1_^b^ [eV]	∆*E*_ST_^c^ [eV]	PLQY^d^ [%]	*τ*_p_^d^ [ns]	*τ*_d_^d^ [µs]	*k*_r_ (10^8^ s^−1^)	*k*_RISC_ (10^4^ s^−1^)
BN-CP	414	430	24	3.10	2.77	0.33	97.0	7.2	105	1.4	1.1

a Measured in dilute toluene solution at 298 K; ^b^Measured in dilute toluene solution at 77 K; ^c^Energy gap of S_1_-T_1_; ^d^Measured in dilute toluene solution after N_2_ bubbling for 10 min at 298 K.

The S_1_ and T_1_ energy levels were determined to be 3.10 eV and 2.77 eV, respectively, by analyzing the fluorescence and phosphorescence spectral onsets. The *ΔE*_ST_ was calculated to be 0.33 eV, facilitating the reverse intersystem crossing (RISC) under ambient conditions, as confirmed by temperature-dependent transient photoluminescence decay studies in deoxygenated solutions (Fig. S7). Compared to BN-CP, intermediate 1 demonstrated distinct photophysical characteristics in dilute toluene solution. It exhibited a broader emission band peaking at 406 nm with a FWHM of 45 nm, accompanied by a significant Stokes shift (Fig. S8). The absorption spectrum of intermediate 1 displayed no fine structure, resembling that of triphenylamine. The *ΔE_ST_* was determined to be 0.53 eV based on the spectral onsets of fluorescence and phosphorescence, substantially larger than that observed in BN-CP. These comparative results reveal that borylation modification effectively narrows the emission spectrum, reduces the Stokes shift, and decreases the singlet–triplet energy gap, ultimately enabling the manifestation of MR-TADF. The prompt (*τ*_p_) and delayed (*τ*_d_) lifetimes of BN-CP at room temperature were measured to be 7.2 ns and 105 µs, respectively. Photophysical rate constants for the fluorescence rate (*k*_r_), the reverse intersystem crossing rate (*k*_RISC_), and the nonradiative transition rate (*k*_nr_) have been further derived from the PLQY and the *τ*_p_/*τ*_d_ ratio using a previously established methodology [32]. These constants were determined to be 1.4 × 10^8^ s^−^^1^, 1.1 × 10^4^ s^−^^1^, and 2.2 × 10^3^ s^−^^1^, respectively. Transient photophysical tests were also conducted on the BN-CP–doped film (1 wt% in a PPF host, Fig. S9), which showed similar results with *k*_r_ of 1.2 × 10^8^ s^−1^, *k*_RISC_ of 1.01 × 10^4^ s^−1^, and *k*_nr_ of 3.0 × 10^3^ s^−1^. The moderate *k*_RISC_ is consistent with the results of previous studies [26,33,34]. These results confirmed the effectiveness of the rigid macrocyclic architecture in reducing vibrational coupling between the ground states and excited states and in minimizing structural relaxation during the radiative process.

### Theoretical calculations

**Figure 5.**
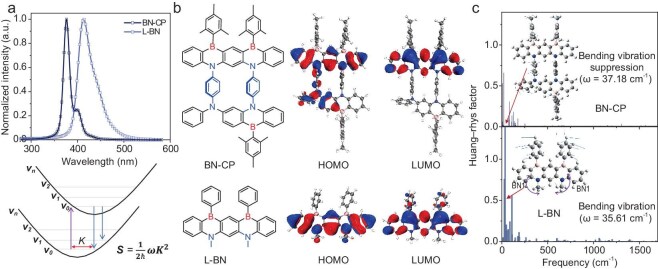
Theoretical comparison of BN-CP and BN2. (a) Simulated photoluminescence spectra of BN-CP and L-BN. (b) HOMO/LUMO distributions of BN-CP and L-BN. (c) Huang–Rhys factors under different vibrational modes of BN-CP and L-BN.

To elucidate the structure–property relationships governing the effect of the macrocyclic architecture on the optoelectronic properties, especially in terms of spectral bandwidth reduction, we have performed extensive quantum mechanical calculations using the density functional theory (DFT) method as well as the time-dependent DFT (TD-DFT) method at the Cam-B3LYP/def2SVP level, utilizing crystallographic data (Fig. 5 and Table S2) [35]. The reported linear analog skeleton L-BN was chosen as a reference [25]. Spectroscopic simulations implementing the Franck–Condon principle revealed that our synthesized cyclophane BN-CP exhibited a significantly reduced FWHM compared to L-BN (16 nm versus 41 nm), which correlated with experimental observations in dilute toluene solutions (Figs 4a, 5a, and Fig. S5). It is well known that the spectral bandwidth is essentially determined by both the structural displacement (*K*) between the S_1_/S_0_ states and the high-frequency vibrational modes [36–39]. Frontier molecular orbital (FMO) analysis, in conjunction with calculated Huang–Rhys factors across multiple vibrational modes, demonstrated that both compounds exhibit comparable high-frequency stretching vibrations with electron density distributions predominantly confined to the L-BN structural framework (Fig. 5b and c). Specifically, the HOMO exhibited significant electron density on nitrogen atoms and adjacent ortho-carbon positions, while the LUMO demonstrated localization on boron atoms and corresponding ortho-carbons. This electronic distribution pattern is typical of MR-TADF systems, which could be further confirmed by hole-charge transfer analysis (Fig. S11). Accordingly, the observed spectral narrowing should be attributed to the increased conformational rigidity after cyclization (Fig. 3a and Fig. S13). According to the analysis, one of the main factors for the broadening of the L-BN spectrum was the bending vibration of the two BN1 planes (vibrational frequency ω: 35.61 cm^−1^, Huang–Rhys factor *S*: 1.39152, reorganization energy λ: 49.55 cm^−1^), which was substantially suppressed in BN-CP (ω: 37.18 cm^−1^, *S*: 0.00495, λ: 0.18 cm^−1^) (Fig. 5c, Fig. S14, and Table S3). Furthermore, the smaller root-mean-square displacement (RMSD) value of BN-CP (0.1031 Å) compared to L-BN (0.1472 Å) further confirms the minimized *K* (Fig. S15). Thus, the macrocyclic framework effectively limited molecular flexibility and suppressed low-frequency vibrational modes, resulting in an exceptionally narrow emission bandwidth.

### Device performance

**Figure 6.**
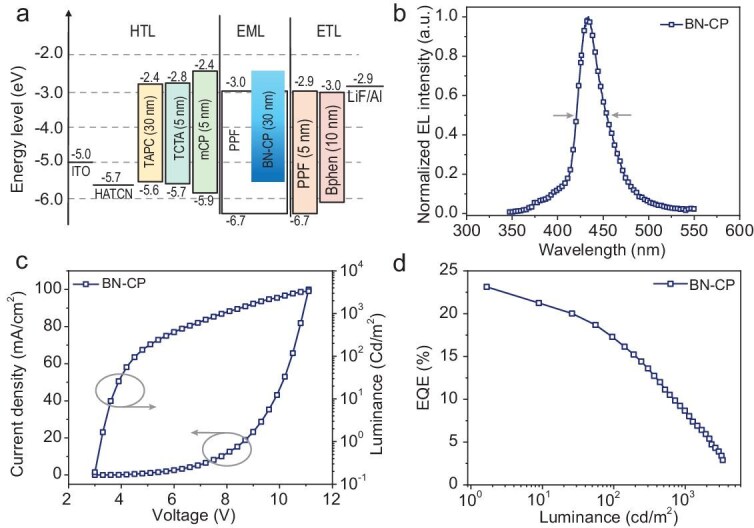
OLED device performance. (a) Device architecture and energy-level diagram of the functional materials for BN-CP-based OLED. (b) The electroluminescence spectrum of BN-CP. (c) Current density and luminance versus voltage (J-V-L) characteristics, and (d) external quantum efficiency-luminance (EQE-L) plots based on BN-CP.

To demonstrate the effectiveness of BN-embedded cyclophane, an OLED was constructed using BN-CP within a carefully optimized architecture: ITO/HATCN (1,4,5,8,9,11-hexaazatriphenylene hexacarbonitrile, 5 nm)/TAPC (1,1-bis[4-[N, N′-di(p-tolyl)amino]phenyl]cyclohexane, 30 nm)/TCTA (4,4′,4′-tris(carbazol-9-yl)triphenylamine, 5 nm)/mCP (1,3-di-9-carbazolylbenzene, 5 nm)/PPF (2,8-bis(diphenylphosphoryl)-dibenzo[b, d]furan): 1 wt% BN-CP (30 nm)/PPF (5 nm)/Bphen (4,7-diphenyl-1,10-phenanthroline, 30 nm)/LiF (0.5 nm)/Al (150 nm). In this configuration, HATCN and LiF served as the hole and electron injection layers, respectively, while TAPC and Bphen functioned as the hole and electron transport layers, respectively. TCTA and PPF were harnessed as electron and hole blocking/hosting materials, respectively. The energy level diagrams and electroluminescence (EL) characteristics of this device are shown in Fig. 6 and Table S4. The molecular structures with the corresponding energy levels of the materials used in the devices are shown in Fig. S16.

The device exhibited a low turn-on voltage (*V*_on_, 1 cd m^−^^2^) of 3.0 V, indicating competent carrier injection and transport processes. EL spectral analysis of BN-CP at 10 mA cm^−^^1^ revealed a deep-blue emission profile with a peak wavelength of 432 nm, a small FWHM of 32 nm, corresponding to a CIE coordinate of (0.16, 0.04) (Fig. 6b), which validates our molecular engineering strategy for spectral narrowing. The observed spectral broadening in EL compared to PL measurements in toluene was attributed to enhanced solid-state molecular interactions and increased polarity of the PPF matrix [12,16,40]. Nevertheless, the device exhibited outstanding performance, achieving an EQE_max_ of 23.3%, which is among the highest for contemporary deep-blue MR-OLEDs with CIEy ≤0.05 (Table S5) [41–45]. Moreover, the EQE_max_ also ranks among the highest reported for macrocyclic TADF emitters, suggesting substantial promise for this molecular class in OLED applications (Fig. S17). The significant efficiency roll-off could be attributed to the inefficient RISC rate of the BN-CP, which could be improved by introducing TADF or phosphorescent sensitizers to increase the *k*_RISC_ of the emitting layer [46–48].

## CONCLUSION

In conclusion, we have successfully synthesized the first narrowband macrocyclic molecule based on the [1,4]azaborine framework via a one-pot triple intramolecular Bora-Friedel–Crafts reaction of aza[1_4_]cyclophane. Structural characterization by X-ray crystallography and DFT simulations demonstrated that the enhanced conformational rigidity induced by cyclization critically enables spectral narrowing. The resulting BN-embedded cyclophane (BN-CP) displays deep-blue emission at 430 nm with a narrow FWHM of 24 nm, which corresponds to a CIE coordinate of (0.17, 0.01). The corresponding OLED device achieves an EQE_max_ of 23.3%, which is among the highest for contemporary deep-blue MR-OLEDs. We are optimistic that the exceptional properties of this novel cyclophane will greatly expand the application scope of macrocyclic TADF molecules.

## METHODS

Both 600 MHz ^1^H NMR and 150 MHz ^13^C-NMR spectra were obtained by a JEOL JNM-ECS600 spectrometer at 298 K in deuterated dichloromethane or chloroform, respectively, with tetramethyl silane as the internal standard. MALDI-TOF-MS data was acquired on a MALDI-TOF instrument (Shimadzu AXIMA Performance) under a positive detection mode. Via the ω-scan mode, diffraction data for single crystal analysis were collected on a Rigaku R-AXIS-RAPID diffractometer, which has been further analyzed and solved using the SHELXTL programs. The corresponding CCDC reference numbers (2 413 115 for BN-CP) and (2 450 282 for intermediate 1) with the data can be obtained from the online Cambridge Crystallographic Data Centre (CCDC) website (https://www.ccdc.cam.ac.uk/structures/).

All DFT and TD-DFT calculations were performed using the Gaussian 16 program [49]. Geometry optimizations as well as the vibration frequency simulation were performed at the CAM-B3LYP/def2SVP level [50–54], in which solvation effects have been taken into consideration, using the CPCM model using cyclohexane as the solvent [55]. The MOMAP software package was used to calculate the absorption/emission spectra and analyze the vibrational modes in the emission spectra based on the simulation data obtained by the Gaussian 16 [56–59]. NTO analysis has been further performed using the Multiwfn package [28].

Details of photophysical tests, fabrication/measurement of electroluminescent devices, and cyclic voltammetry measurements are provided in the Supplementary Data.

## Supplementary Material

nwaf250_Supplemental_File
