# Supplementary material for: Narrowband deep-blue emission from a BN-embedded cyclophane: synthesis, characterization, and OLED application
Source: Natl Sci Rev. 2025 Jul 3;12(8):nwaf250. doi: 10.1093/nsr/nwaf250 (PMC12260501; doi:10.1093/nsr/nwaf250)
Supplement: nwaf250_Supplemental_File [file nwaf250_supplemental_file.pdf]

**RESEARCH ARTICLE**

**MATERIALS SCIENCE**

# Narrowband deep-blue emission from a BN-embedded cyclophane: synthesis, characterization, and OLED application

Tianjiao Fan,[1] Cheng Qu,[1] Lian Duan,[1,2,*] and Yuewei Zhang[2,*]

[1] Key Lab of Organic Optoelectronics and Molecular Engineering of Ministry of Education, Department of Chemistry, Tsinghua University, Beijing 100084, China

[2] Laboratory of Flexible Electronics Technology, Tsinghua University, Beijing, 100084, China;

∗**Corresponding authors.** E-mails: duanl@mail.tsinghua.edu.cn; zhangyuewei@mail.tsinghua.edu.cn

## Experimental Section

### Synthesis

N1,N4-diphenylbenzene-1,4-diamine, 1,3-dibromobenzene, $Pd_2(dba)_3$ (tris (dibenzylideneacetonyl) bis-palladium), X-Phos (2-(Dicyclohexylphosphino) -2',4',6' -tri-i-propyl-1,1'-biphenyl), t-BuONa were purchased from Bide Pharmatech Ltd.. N,N-Diisopropylethylamine (DIPEA), Boron tribromide, as well as Bromo(2,4,6-trimethylphenyl)magnesium (MesMgBr, 1M in THF) was purchased from Energy Chemical. Petroleum ether, $CH_2Cl_2$, , xylene and o-dichlorobenzene (o-DCB) were purchased from Sinopharm Chemical Reagent Co., Ltd..

### Synthesis of 2,4,6,8-tetraphenyl-2,4,6,8-tetraaza-1,5(1,3),3,7(1,4)-tetrabenzenacyclooctaphane (intermediate 1)

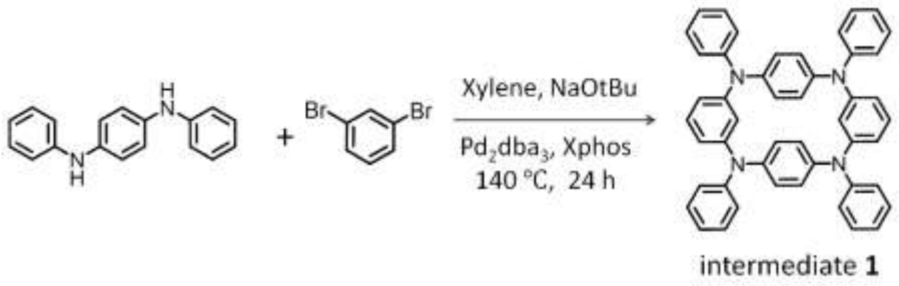


$N^1$,$N^4$-diphenylbenzene-1,4-diamine (4.0 g, 15.4 mmol), 1,3-dibromobenzene (3.62 g, 15.4 mmol), $Pd_2(dba)_3$ (703 mg, 0.768 mmol, 0.05 eq), X-Phos (366 mg, 0.768 mmol, 0.05 eq) and t-BuONa (8.86 g, 92.2

mmol, 6 eq) were dissolved in xylene (200 mL) at room temperature. The mixture was stirred at 140 ℃ under nitrogen atomoshpere for 24 h. Then the reaction mixture was poured into a large amount of water. Then the product was extracted with dichloromethane. After filtration and evaporation, the crude product was purified by recrystallization from toluene/methanol to afford intermediate **1** as a grey solid. $^{1}$H NMR (600 MHz, Methylene Chloride-$d_2$) δ 7.19 – 7.16 (m, 8H), 7.12 – 7.09 (m, 8H), 6.95 – 6.91 (m, 6H), 6.78 (s, 8H), 6.55 (dd, J = 8.0, 2.2 Hz, 4H), 6.23 (t, J = 2.3 Hz, 2H). $^{13}$C NMR (151 MHz, Methylene Chloride-$d_2$) δ 149.07, 147.11, 143.01, 129.19, 129.12, 126.02, 124.42, 122.80, 116.78, 115.15. MALDI-TOF: Calculated: 668.84, Found: 668.2293.

**Synthesis of BN-CP**

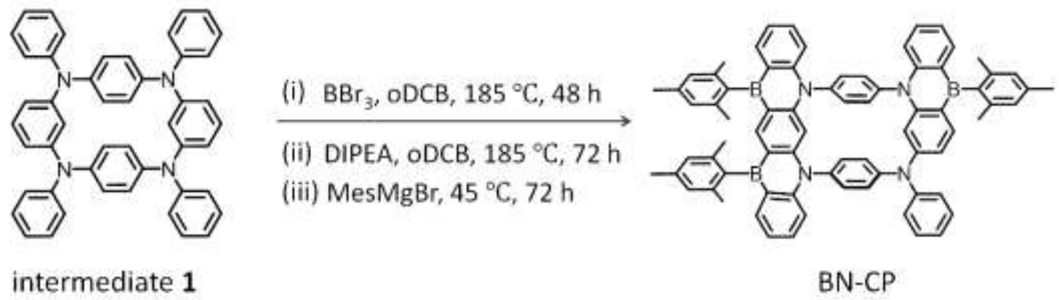


Boron tribromide (1.7 mL, 17.9 mmol) was added was added slowly to a solution of compound **1** (1.0 g, 1.50 mmol) in o-dichlorobenzene (60 mL) in a high-pressure flask at 0 ℃ under a nitrogen atmosphere. After stirring at 185 ℃ for 2 days, N, N-Diisopropylethylamine (3.9 mL, 22.4 mmol) was

added at 0 ℃ and the reaction mixture was allowed to warm to room temperature. After stirring at 185 ℃ for 3 days, Bromo(2,4,6-trimethylphenyl)magnesium in THF solution (1 M, 30 mL) was added at 0 ℃ and the reaction mixture was stirred at 45 ℃ for 3 days. The reaction mixture was quenched by ethanol and the mixture was evaporated under reduced pressure. The crude product was purified by column chromatography on a silica gel (petroleum ether/ dichloromethane= 9:1 v/v) and recrystallized from dichloromethane and acetonitrile as a faint yellow powder (0.23 g, yield: 14.7%). $^{1}$H NMR (600 MHz, Methylene Chloride-$d_2$) δ 8.38 (s, 1H), 7.72 (d, J = 7.3 Hz, 1H), 7.70 – 7.67 (m, 1H), 7.64 (d, J = 2.5 Hz, 5H), 7.59 (s, 1H), 7.54 (dq, J = 6.4, 2.1 Hz, 2H), 7.42 (ddd, J = 11.4, 8.5, 2.2 Hz, 3H), 7.35 – 7.30 (m, 4H), 7.28 (dd, J = 8.5, 2.4 Hz, 2H), 7.16 – 7.13 (m, 1H), 7.09 – 7.06 (m, 2H), 7.00 (dt, J = 7.1, 3.4 Hz, 1H), 6.91 (s, 2H), 6.88 (d, J = 5.0 Hz, 1H), 6.83 (d, J = 6.4 Hz, 5H), 6.78 – 6.75 (m, 1H), 6.69 – 6.63 (m, 1H), 6.51 (s, 1H), 6.37 (s, 1H), 2.33 (s, 3H), 2.30 (d, J = 3.8 Hz, 6H), 2.02 (s, 6H), 1.93 (s, 6H), 1.91 (s, 6H). $^{13}$C NMR (151 MHz, Methylene Chloride-$d_2$) δ 152.35, 151.27, 150.01, 149.87, 148.05, 147.36, 147.30, 146.82, 146.47, 146.03, 141.93, 141.70, 139.13, 139.00, 138.29, 137.85, 137.09, 136.87, 136.63, 136.57, 136.55, 136.25, 133.31, 133.21, 133.03, 132.95, 132.38, 131.58, 130.70, 129.68, 129.61, 126.86, 126.78, 126.73, 126.67, 126.48, 125.10, 120.38, 120.19, 119.97, 116.81, 116.41,

112.15, 105.06, 101.74, 22.99, 22.93, 20.95. MALDI-TOF: Calculated: 1052.79, Found: 1052.1672.

**Photophyical test**

The concentration of the dilute solution ($1\times10^{-5}$ M) was prepared by stepwise dilution for solution state measurements. Doped films for photophysical characterization were prepared by thermal evaporation on quartz substrates at 1-2 Å $sec^{-1}$ in a vacuum chamber with a base pressure of < $10^{-5}$ torr. UV-vis absorption and PL spectra were measured using UV-2600 (Shimadzu) and FluoroMax-4P (Horiba) instruments. The quantum yields were obtained with an absolute photoluminescence quantum yield (PLQY) measurement system Hamamatsu C9920-03G. The solution samples were bubbled with nitrogen for 10 minutes and then sealed before the PLQY and delayed transient spectral measurement while the films were measured under air atmosphere. The transient spectra of solution samples and film samples were all obtained via an Edinburgh Fluorescence Spectroscopy FLS1000.

**Fabrication/measurement of electroluminescent devices**

All compounds were subjected to temperature-gradient sublimation under high vacuum before use. OLEDs were fabricated on indium-tin-

oxide (ITO)-coated glass substrates, with multiple organic layers sandwiched between the transparent bottom ITO anode and the top aluminum cathode. Prior to device fabrication, the ITO glass substrates were first washed multiple times with distilled water and absolute ethanol, and dried using an infrared lamp.

All material layers were deposited by vacuum evaporation in a vacuum chamber with a base pressure of $10^{-6}$ torr. The deposition system enables the fabrication of the complete device structure in a single vacuum pump-down without breaking the vacuum. The deposition rate of the organic layers was maintained at 0.1–0.2 nm/s. Doping was performed by co-evaporation from separate evaporation sources with different evaporation rates.

The current density, voltage, luminance, EQE, electroluminescent spectra, and other characteristics were measured simultaneously using a Keithley 2400 source meter and an absolute EQE measurement system in an integrating sphere. The EQE measurement system is a Hamamatsu C9920-12 equipped with a Hamamatsu PMA-12 photonic multichannel analyzer C10027-02, which has a maximum detection wavelength of 1100 nm.

**Cyclic voltammetry**

Cyclic voltammetry was conducted on a CHI 660 instrument, with a platinum (Pt) electrode serving as the working electrode, a Pt wire as the auxiliary electrode, and an $Ag/Ag^{+}$ electrode as the reference electrode. The oxidation/reduction potentials were measured in dry dichloromethane/DMF solutions containing 0.1 M tetrabutylammonium hexafluorophosphate ($TBAPF_6$) as the supporting electrolyte at a scan rate of 100 mV $s^{-1}$. The potentials are based on the ferrocene/ferrocenium couple. The HOMO and LUMO levels were approximately estimated from the oxidation/reduction potentials:

$E_{HOMO} = - (E_{[onset,ox\ vs.\ Fc^{+}/Fc]} + 4.8)$ (eV)   $E_{LUMO} = - (E_{[onset,red\ vs.\ Fc^{+}/Fc]} + 4.8)$ (eV)

**Table S1.** Crystal Data and Structure Refinement of BN-CP.

| | BN-CP |
|---|---|
| empirical formula | C79 H65 B3 Cl2 N4 |
| formula wt | 1173.68 |
| crystal system | orthorhombic |
| *T* (K) | 169.99(10) |
| space group | F d d 2 |
| *a*/Å | 33.8830(7) |
| *b*/Å | 102.868(2) |
| *c*/Å | 7.43860(10) |
| *α*/ ° | 90 |
| β/ ° | 90 |
| *γ*/ ° | 90 |
| *V*/Å3 | 25927.1(8) |
| *Z* | 16 |
| density, g/$cm^3$ | 1.203 |
| *F (000)* | 9856 |
| θ range/Å | 1.718 to 77.763 |

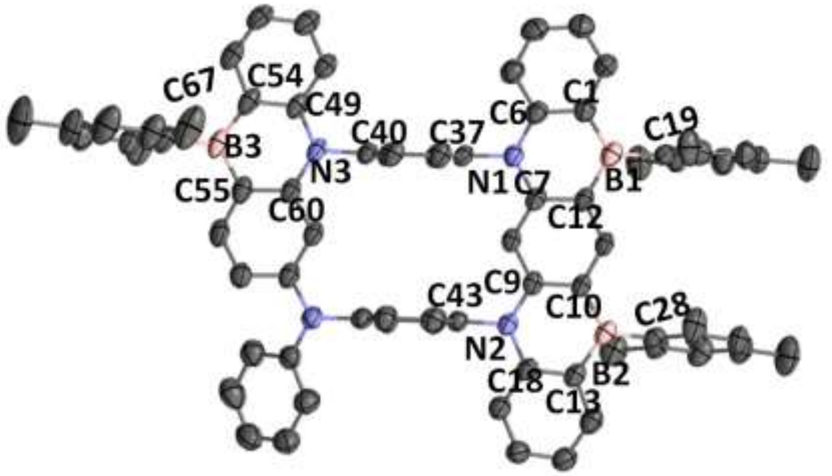


| | |
|---|---|
| B1-C1: 1.515(6) Å | N1-C6: 1.404(6) Å |
| B1-C12: 1.521(7) Å | N1-C7: 1.407(5) Å |
| B1-C19: 1.594(7) Å | N1-C37: 1.440(5) Å |
| B2-C10: 1.546(6) Å | N2-C9: 1.387(6) Å |
| B2-C13: 1.525(7) Å | N2-C18: 1.413(5) Å |
| B2-C28: 1.563(6) Å | N2-C43: 1.450(5) Å |
| B3-C54: 1.507(8) Å | N3-C40 : 1.454(5) Å |
| B3-C55: 1.519(6) Å | N3-C49: 1.396(6) Å |
| B3-C67: 1.600(7) Å | N3-C60: 1.374(6) Å |

**Figure. S1.** ORTEP drawing of BN-CP obtained by X-ray crystallographic analysis. Thermal ellipsoids are shown at 50% probability.

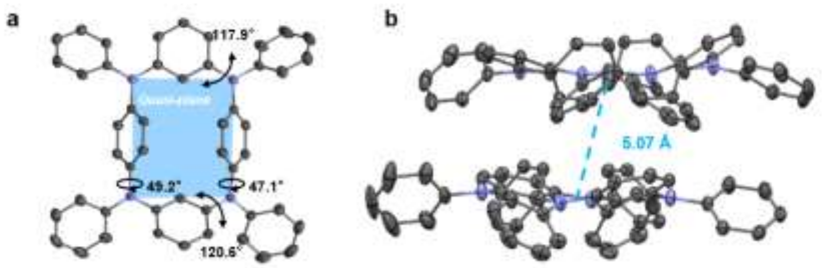


**Figure S3.** Single-crystal structures of intermediate **1** with 50% thermal ellipsoid probability. (a) Selected angles between the central bridging benzene ring and the diphenylamine fragments and (b) distance between two neighboring molecules under packing mode.

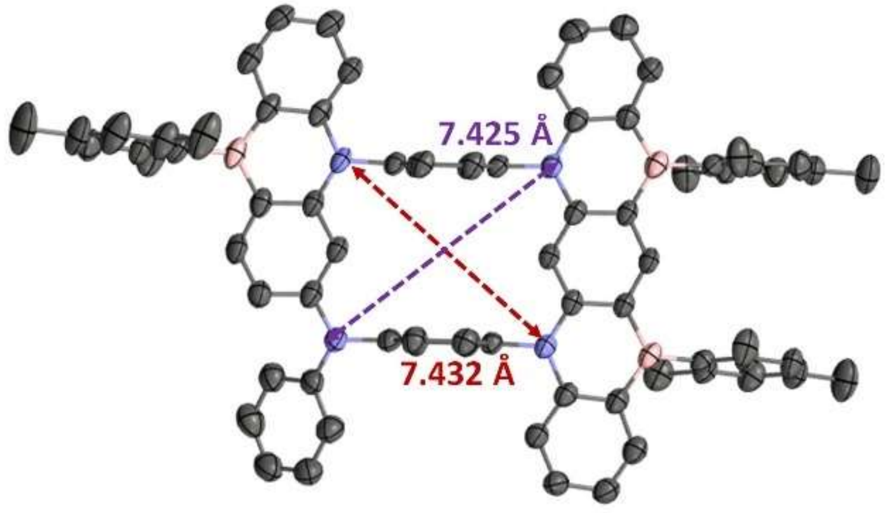


**Figure. S2.** The cavity diameter of BN-CP crystal.

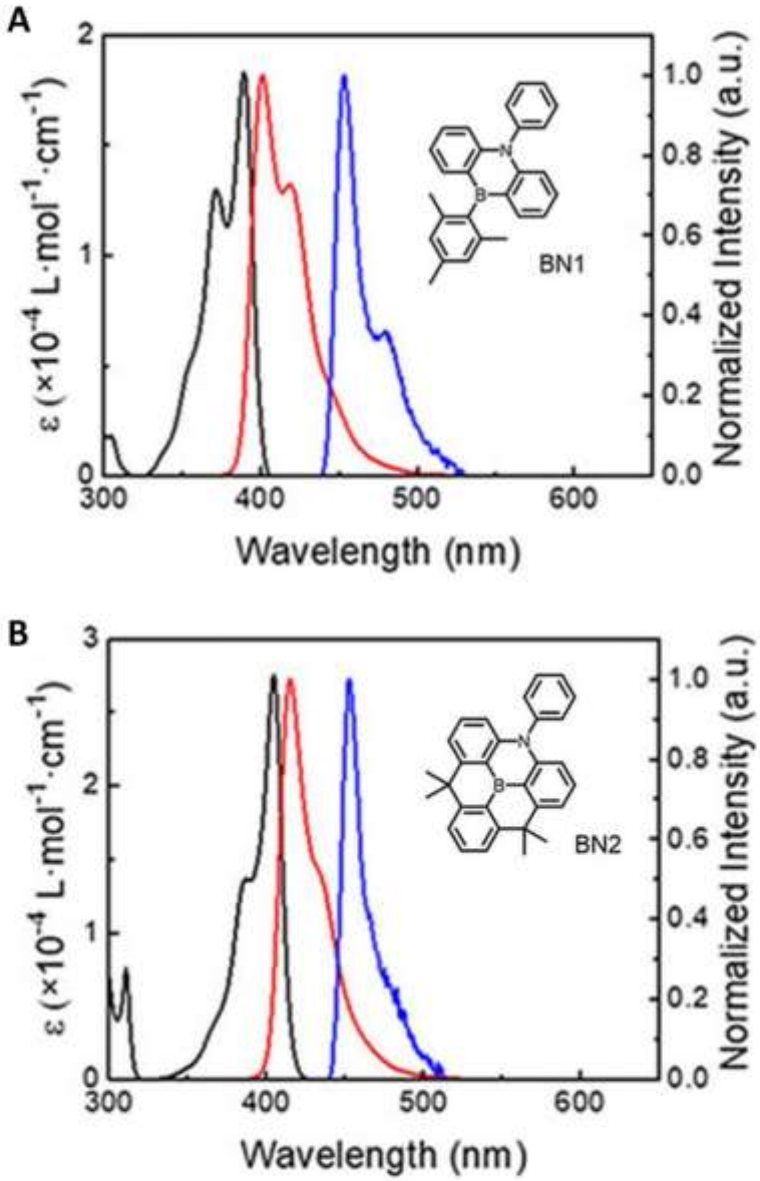


**Figure. S4.** Absorption (black line), fluorescence (red line), and phosphorescence (blue line, at 77 K) spectra of BN1 (A) and BN2 (B) in toluene (1.0 × 10–5 mol L−1). Adapted with permission (51). Copyright 2022, Frontiers.

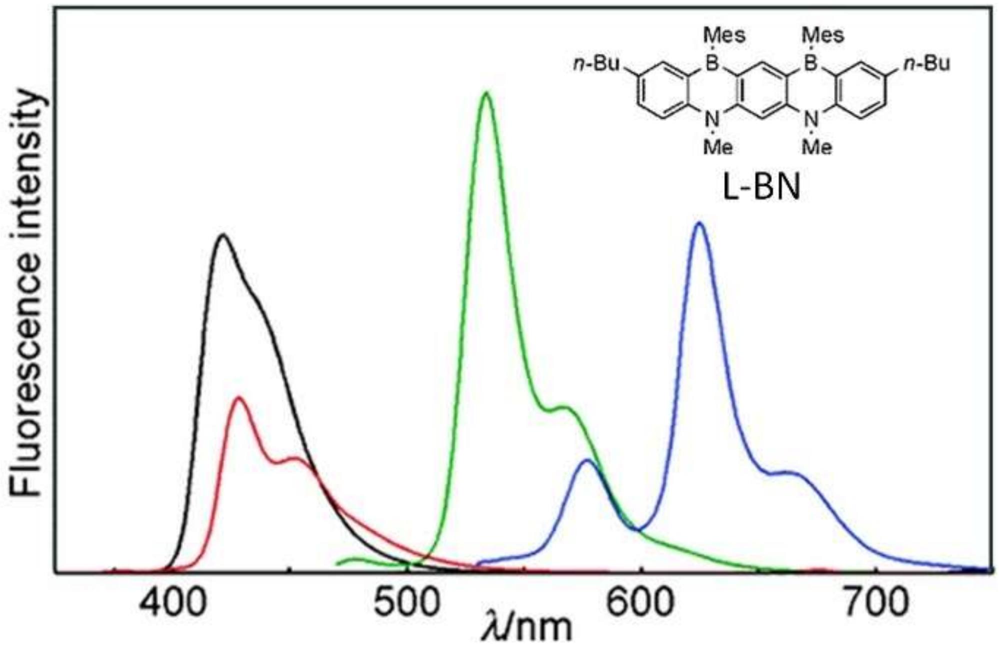


**Figure. S5**. Fluorescence spectra of L-BN in cyclohexane at 298 K (red line) (*25*). Copyright 2006, American Chemical Society.

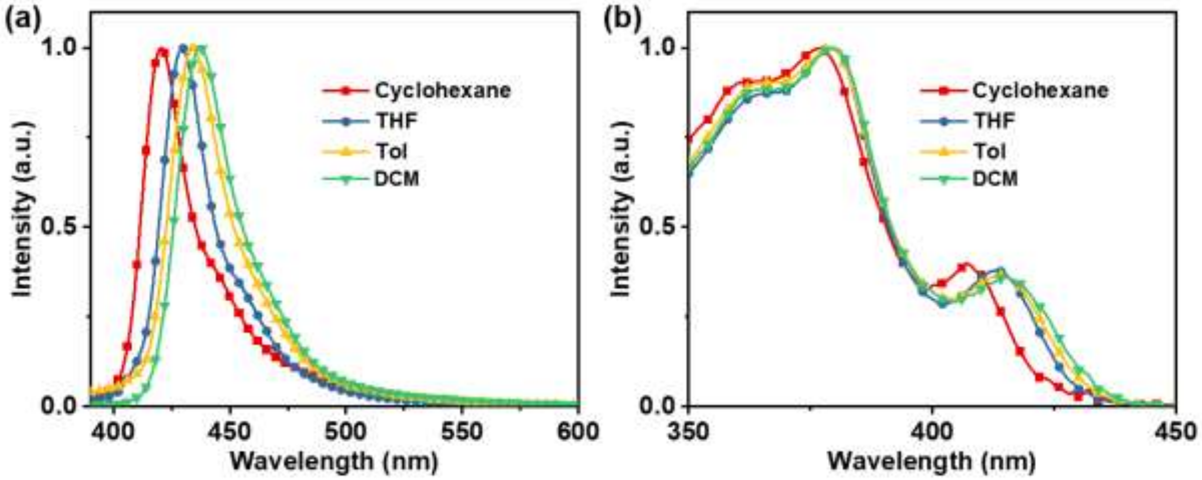


**Figure. S6**. Solvatochromism effect of BN-CP on fluorescence spectra (a) and absorption spectra (b) in different solvents.

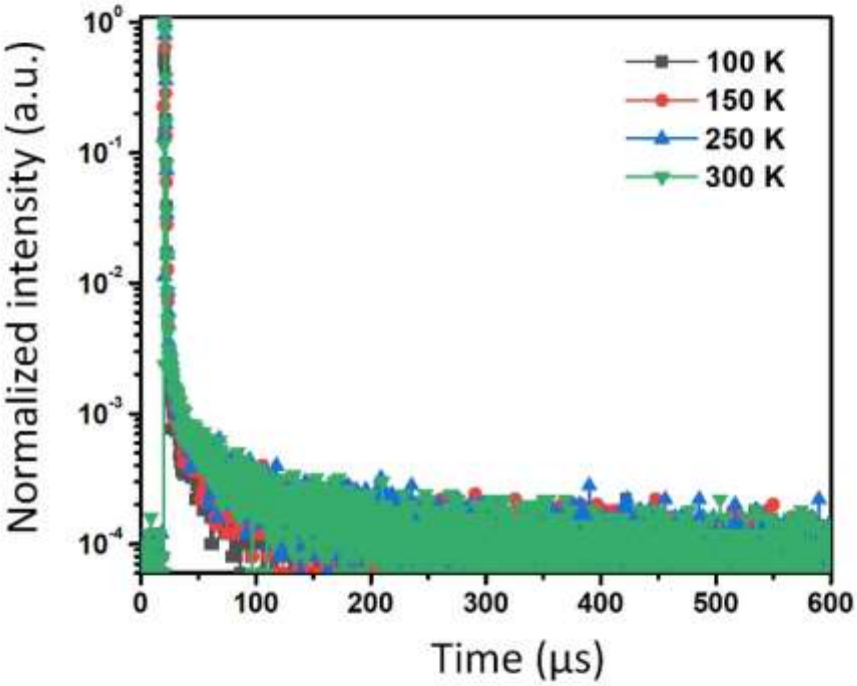


**Figure. S7**. Transient decay spectra of BN-CP in toluene solution at 100-300 K.

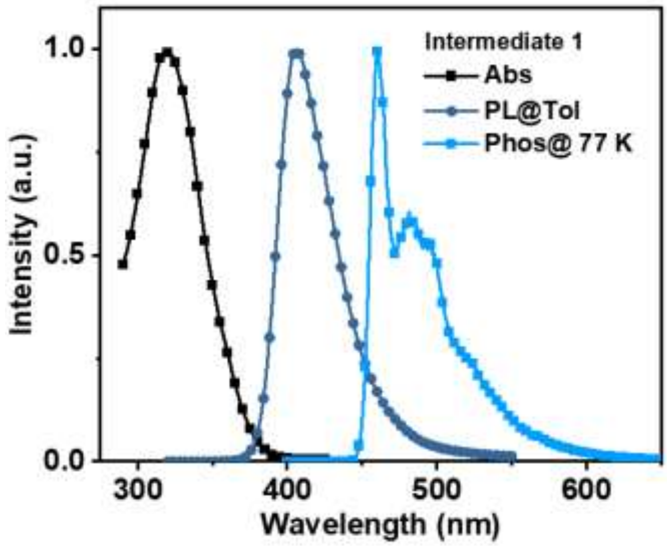


**Figure S8.** Normalized UV-vis absorption, fluorescence and phosphorescence (under 77 K) spectra of intermediate **1** in toluene.

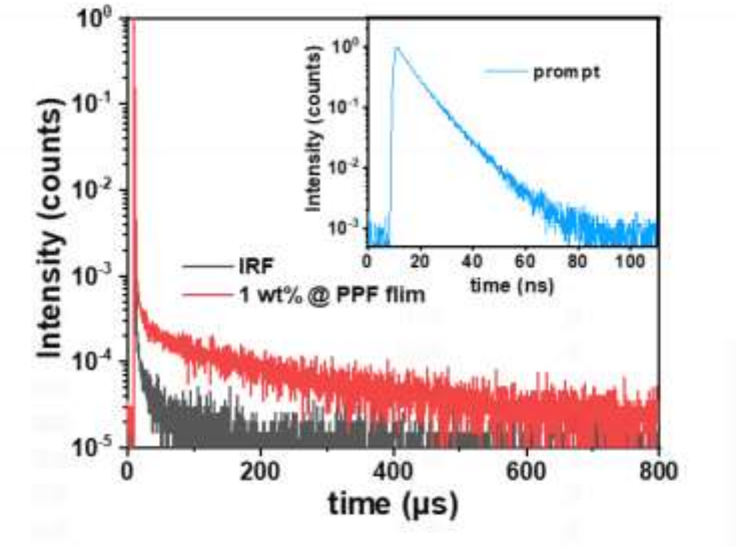


**Figure S9.** Transient photoluminescence decay curves of BN-CP in PPF films with 1 wt% doping concentration.

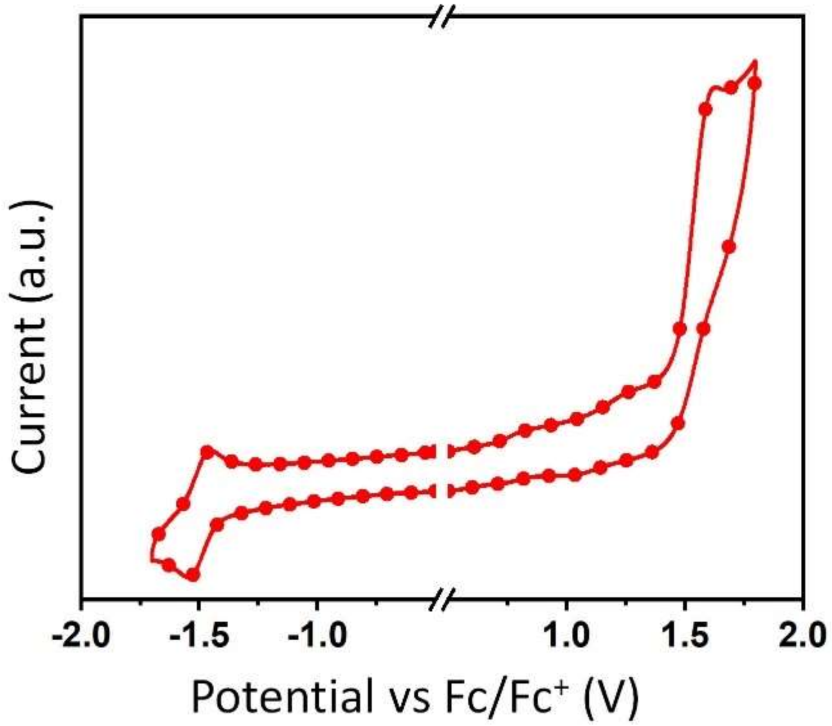


**Figure. S10**. Cyclic voltammograms of BN-CP.

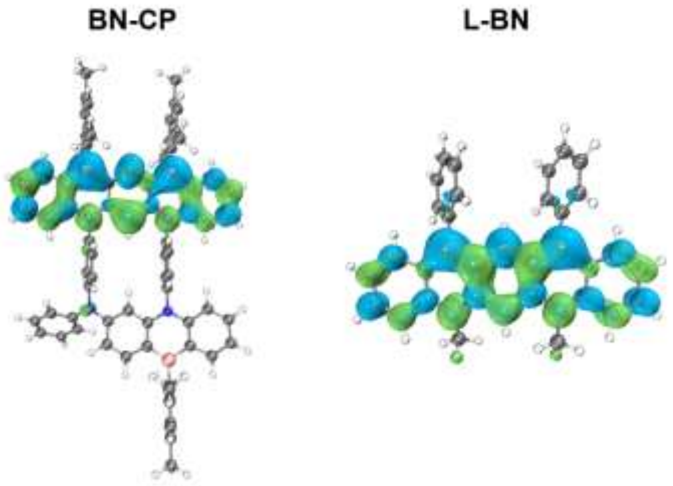


**Figure. S11.** Charge density difference between the ground state and the excited states of BN-CP and L-BN with the density increase (green) and density decrease (blue).

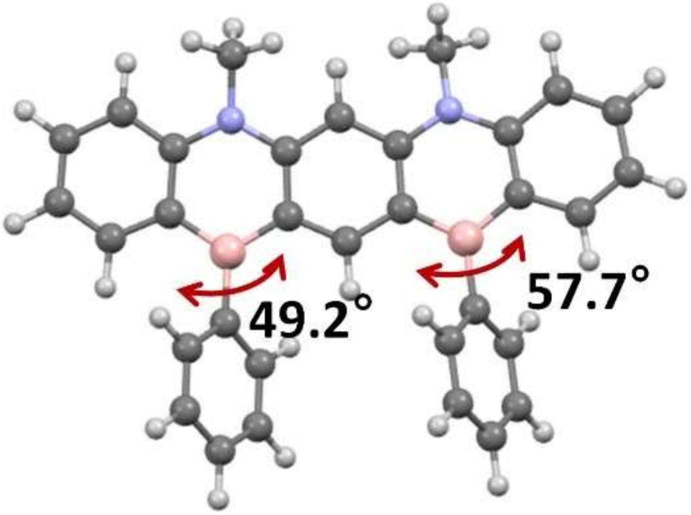


**Figure. S12**. Theoretical optimized planar structure of L-BN. The result was calculated at the cam-B3LYP/6-31G (d) level.

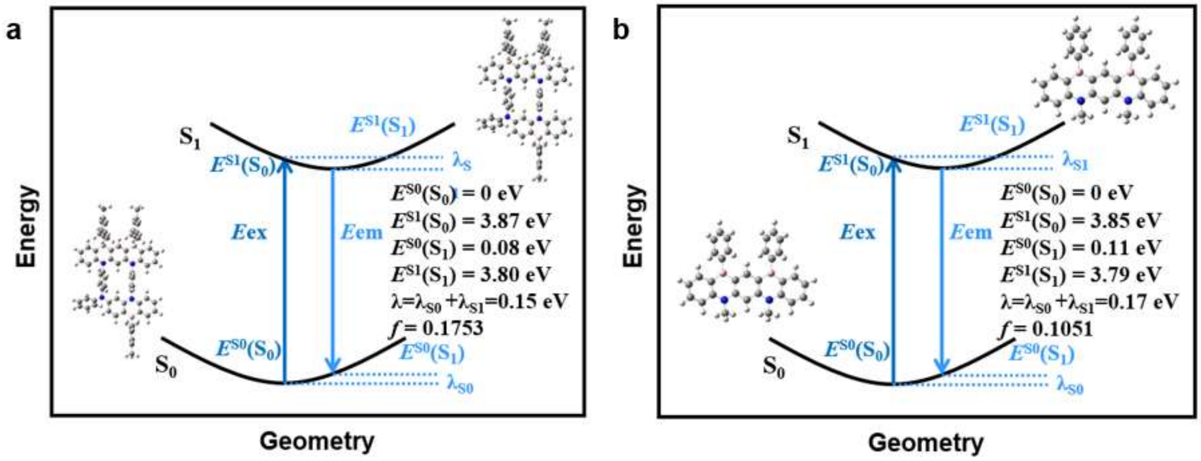


**Figure. S13.** Reorganization energy comparison. Optimized $S_0$ and $S_1$ structures, reorganization ($\lambda_S$) and structural relaxation ($\lambda_S$*) energies of BN-CP (a) and L-BN (b).

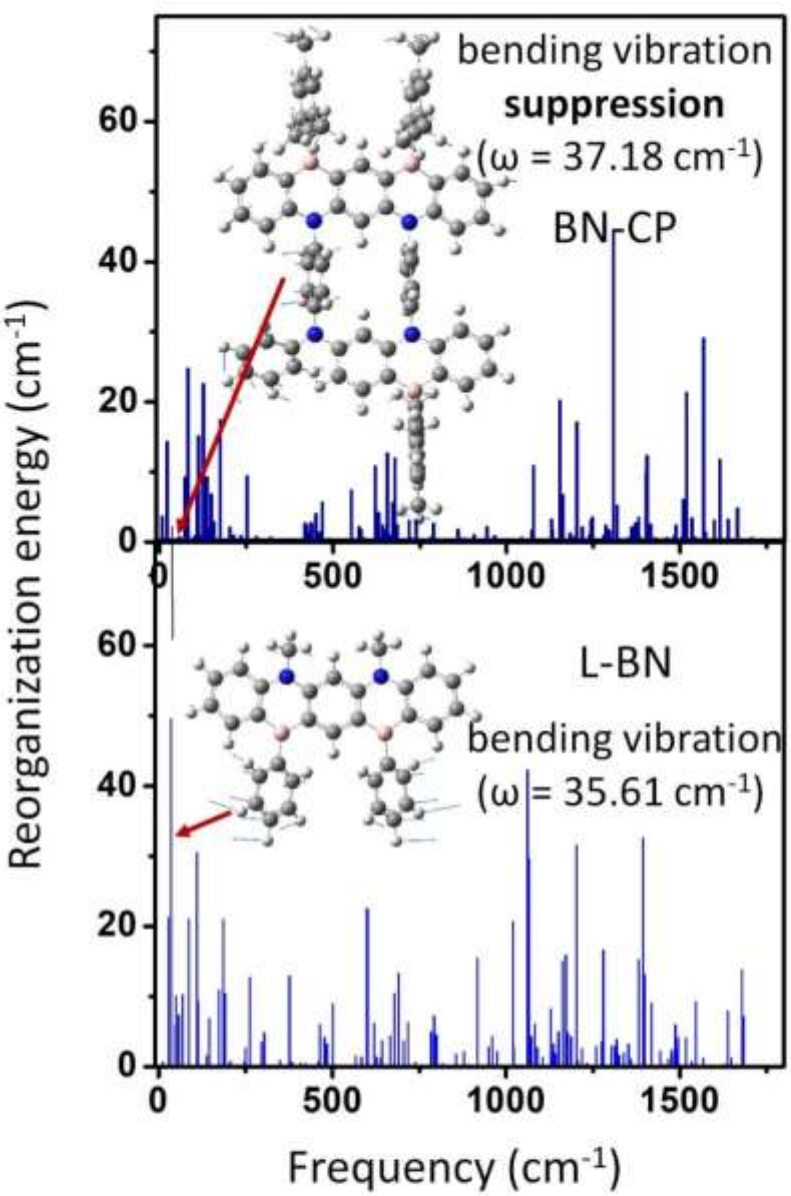


**Figure. S14.** Reorganization energy comparison under different vibrational modes. The reorganization energies under different vibrational modes of BN-CP and L-BN.

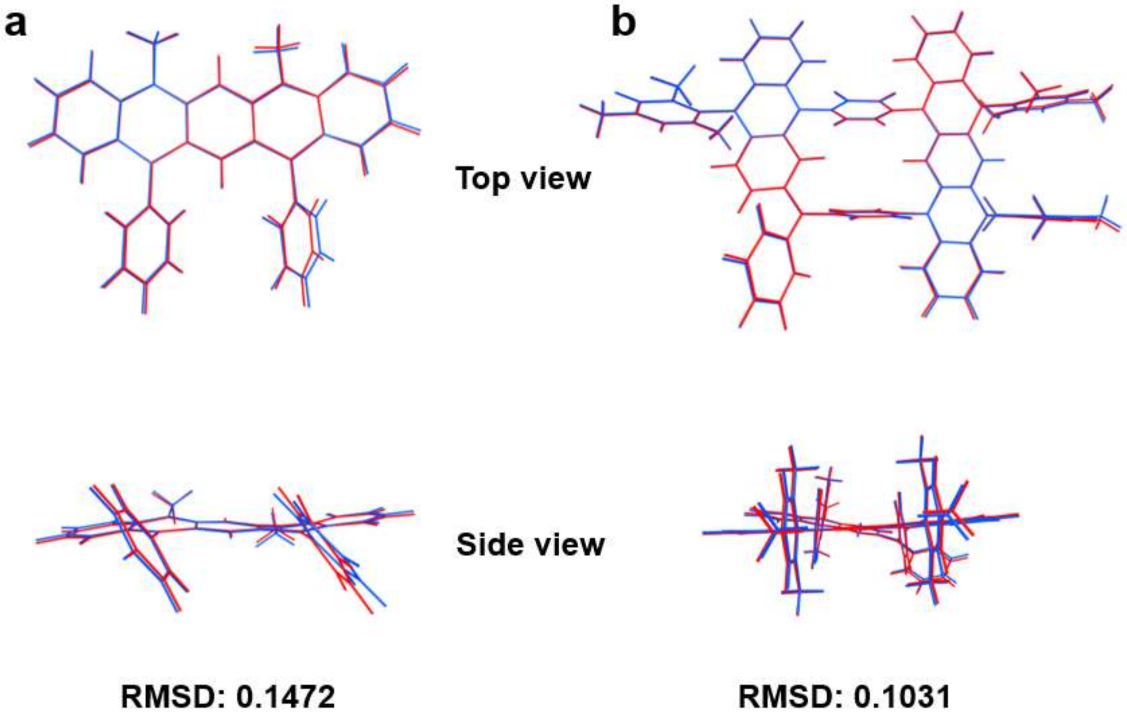


**Figure. S15.** Root-mean-square displacement analysis. Comparison of the optimized structures of BN-CP (a) and L-BN (b) in the $S_0$ (blue) and $S_1$ (red) states.

**Table S2.** Summary of TD-DFT calculations for BD-CP and L-CP at the $S_0$ and $S_1$ structures at the cam-B3LYP/6-31G (d) level.

| Compound | Optimized Structure | Transition | Wavelength (nm) | Energy (eV) | Oscillator Strength |
|---|---|---|---|---|---|
| BN-CP | $S_0$ | $S_0 \rightarrow S_1$ | 320.47 | 3.8688 | 0.1765 |
| | | $S_0 \rightarrow T_1$ | 415.85 | 2.9815 | 0.0000 |
| | $S_1$ | $S_1 \rightarrow S_0$ | 336.66 | 3.6827 | 0.1051 |
| | $T_1$ | $T_1 \rightarrow S_0$ | 513.49 | 2.4145 | 0.0000 |
| L-CP | $S_0$ | $S_0 \rightarrow S_1$ | 277.59 | 4.4665 | 0.2680 |
| | | $S_0 \rightarrow T_1$ | 411.01 | 3.0166 | 0.0000 |
| | $S_1$ | $S_1 \rightarrow S_0$ | 336.66 | 3.6827 | 0.1051 |
| | $T_1$ | $T_1 \rightarrow S_0$ | 460.81 | 2.6905 | 0.0000 |

**Table S3.** All vibrational modes in the $S_0$ state involved in the Franck-Condon spectral progression of BO-DICz and TPABO-DICz at the cam-B3LYP/6-31G (d) level (in cyclohexane).

| | BN-CP | | | | L-BN | | |
|---|---|---|---|---|---|---|---|
| Mode | Freq | HRhys | Reorg | Mode | Freq | HRhys | Reorg |
| 1 | 5.74 | 0.0014 | 0.01 | 1 | 13.14 | 0.04804 | 0.63 |
| 2 | 6.97 | 0.52923 | 3.69 | 2 | 28.04 | 0.75754 | 21.24 |
| 3 | 9.42 | 0.06083 | 0.57 | 3 | 35.61 | 1.39152 | 49.55 |
| 4 | 9.77 | 0.00108 | 0.01 | 4 | 48.44 | 0.12265 | 5.94 |
| 5 | 13.24 | 0.02231 | 0.3 | 5 | 49.36 | 0.20326 | 10.03 |
| 6 | 14.47 | 0.01443 | 0.21 | 6 | 57.48 | 0.12715 | 7.31 |
| 7 | 16.91 | 0.00111 | 0.02 | 7 | 62.45 | 0.00134 | 0.08 |
| 8 | 20.8 | 0.01733 | 0.36 | 8 | 69.53 | 0.14699 | 10.22 |
| 9 | 21.77 | 0.65808 | 14.33 | 9 | 87.29 | 0.24075 | 21.02 |
| 10 | 22.18 | 0.00602 | 0.13 | 10 | 109.56 | 0.55654 | 60.97 |
| 11 | 24.57 | 5.3E-4 | 0.01 | 11 | 112.68 | 0.08243 | 9.29 |
| 12 | 25.7 | 0.00245 | 0.06 | 12 | 138.29 | 0.01069 | 1.48 |
| 13 | 28.98 | 0.00159 | 0.05 | 13 | 145.93 | 0.04645 | 6.78 |
| 14 | 31.52 | 5.8E-4 | 0.02 | 14 | 174.21 | 0.06249 | 10.89 |
| 15 | 37.18 | 0.00495 | 0.18 | 15 | 178.96 | 0 | 0 |
| 16 | 40.58 | 0.01062 | 0.43 | 16 | 187.2 | 0.11177 | 20.92 |
| 17 | 48.01 | 0.00261 | 0.13 | 17 | 192.06 | 0.05402 | 10.37 |
| 18 | 56.59 | 0.01293 | 0.73 | 18 | 200.46 | 0.0022 | 0.44 |
| 19 | 57.2 | 3E-5 | 0 | 19 | 208.52 | 0.00383 | 0.8 |
| 20 | 61.14 | 0.0073 | 0.45 | 20 | 223.88 | 2E-5 | 0.01 |
| 21 | 63.39 | 9.9E-4 | 0.06 | 21 | 247.36 | 0.00259 | 0.64 |
| 22 | 66.15 | 3E-5 | 0 | 22 | 250.24 | 0.01037 | 2.59 |
| 23 | 71.67 | 0 | 0 | 23 | 263.19 | 0.04828 | 12.71 |
| 24 | 72.15 | 0.12758 | 9.21 | 24 | 296.46 | 0.01154 | 3.42 |
| 25 | 81.31 | 0.30493 | 24.79 | 25 | 304.14 | 0.01578 | 4.8 |
| 26 | 81.58 | 0.08421 | 6.87 | 26 | 349.43 | 0.00246 | 0.86 |
| 27 | 89.44 | 0.00182 | 0.16 | 27 | 377.09 | 0.03408 | 12.85 |
| 28 | 90.19 | 1.1E-4 | 0.01 | 28 | 383.87 | 0.00155 | 0.59 |

| | | | | | | | |
|---|---|---|---|---|---|---|---|
| 29 | 98.26 | 0.0014 | 0.14 | 29 | 408.3 | 0.00117 | 0.48 |
| 30 | 100.55 | 0.00878 | 0.88 | 30 | 420.44 | 1E-5 | 0.01 |
| 31 | 108.51 | 0.05916 | 6.42 | 31 | 422.63 | 9.5E-4 | 0.4 |
| 32 | 108.96 | 0.00221 | 0.24 | 32 | 430.39 | 2.6E-4 | 0.11 |
| 33 | 109.91 | 0.00288 | 0.32 | 33 | 432.58 | 2E-5 | 0.01 |
| 34 | 111.09 | 0.13621 | 15.13 | 34 | 458.78 | 0.00145 | 0.66 |
| 35 | 118.44 | 0.00714 | 0.85 | 35 | 463.86 | 0.01301 | 6.03 |
| 36 | 121.17 | 3E-4 | 0.04 | 36 | 477.57 | 0.00865 | 4.13 |
| 37 | 125.36 | 0.18008 | 22.57 | 37 | 484.8 | 0.00652 | 3.16 |
| 38 | 132.35 | 0.01149 | 1.52 | 38 | 501.04 | 0.01782 | 8.93 |
| 39 | 134.62 | 0.04433 | 5.97 | 39 | 517.04 | 2.8E-4 | 0.15 |
| 40 | 136.12 | 0.06789 | 9.24 | 40 | 543.38 | 0 | 0 |
| 41 | 143.14 | 0.01219 | 1.74 | 41 | 555.67 | 1.1E-4 | 0.06 |
| 42 | 146.74 | 0.01381 | 2.03 | 42 | 566.86 | 0.00257 | 1.45 |
| 43 | 148.43 | 0.04592 | 6.82 | 43 | 583.57 | 0.00222 | 1.29 |
| 44 | 155.35 | 0.01824 | 2.83 | 44 | 599.09 | 0.03759 | 22.52 |
| 45 | 175.41 | 0.09956 | 17.46 | 45 | 602.12 | 0.03725 | 22.43 |
| 46 | 190.22 | 3E-5 | 0.01 | 46 | 618.61 | 0 | 0 |
| 47 | 190.66 | 3E-5 | 0.01 | 47 | 620.45 | 0.00987 | 6.13 |
| 48 | 192.74 | 4.9E-4 | 0.09 | 48 | 628.16 | 0.00208 | 1.3 |
| 49 | 202.45 | 0.01043 | 2.11 | 49 | 639.1 | 0.00176 | 1.13 |
| 50 | 213.11 | 0.00425 | 0.9 | 50 | 642.46 | 0.00569 | 3.65 |
| 51 | 217.74 | 2E-5 | 0 | 51 | 665.48 | 0.00659 | 4.39 |
| 52 | 221.29 | 2E-5 | 0.01 | 52 | 678.71 | 0.01533 | 10.4 |
| 53 | 224.41 | 2.2E-4 | 0.05 | 53 | 690.15 | 0.01933 | 13.34 |
| 54 | 227.04 | 3.2E-4 | 0.07 | 54 | 704.92 | 0.00515 | 3.63 |
| 55 | 234.85 | 0.00362 | 0.85 | 55 | 717.72 | 0.00876 | 6.29 |
| 56 | 236.39 | 3E-5 | 0.01 | 56 | 735.4 | 3E-5 | 0.03 |
| 57 | 236.74 | 3E-5 | 0.01 | 57 | 738.03 | 7.5E-4 | 0.55 |
| 58 | 237.36 | 1.9E-4 | 0.05 | 58 | 739.33 | 4E-4 | 0.3 |
| 59 | 252.63 | 0.0372 | 9.4 | 59 | 751.74 | 7E-5 | 0.05 |
| 60 | 259.56 | 0.00177 | 0.46 | 60 | 778.86 | 1.7E-4 | 0.13 |

| | | | | | | | |
|---|---|---|---|---|---|---|---|
| 61 | 270.84 | 1E-5 | 0 | 61 | 783.66 | 0.00622 | 4.88 |
| 62 | 277.61 | 3.6E-4 | 0.1 | 62 | 792.66 | 0.00908 | 7.2 |
| 63 | 278.66 | 0.00271 | 0.75 | 63 | 795.19 | 0.00439 | 3.49 |
| 64 | 278.99 | 0 | 0 | 64 | 799.31 | 0.00556 | 4.44 |
| 65 | 279.12 | 1.5E-4 | 0.04 | 65 | 817.25 | 5.1E-4 | 0.42 |
| 66 | 292.02 | 3.8E-4 | 0.11 | 66 | 856 | 0.00202 | 1.73 |
| 67 | 301.58 | 4.9E-4 | 0.15 | 67 | 866.46 | 2E-4 | 0.18 |
| 68 | 309.67 | 6.5E-4 | 0.2 | 68 | 879.03 | 0.0024 | 2.11 |
| 69 | 317.25 | 8E-5 | 0.02 | 69 | 884.54 | 6E-5 | 0.05 |
| 70 | 320.54 | 0.00214 | 0.68 | 70 | 891.26 | 3.2E-4 | 0.28 |
| 71 | 329.69 | 2.3E-4 | 0.07 | 71 | 892.37 | 0 | 0 |
| 72 | 332.92 | 4.3E-4 | 0.14 | 72 | 903.29 | 1.8E-4 | 0.16 |
| 73 | 348.8 | 5.6E-4 | 0.2 | 73 | 917.59 | 0.01682 | 15.43 |
| 74 | 352.42 | 7.3E-4 | 0.26 | 74 | 925.77 | 4E-5 | 0.04 |
| 75 | 377.88 | 1E-5 | 0 | 75 | 949.31 | 0.00287 | 2.72 |
| 76 | 388.38 | 6E-5 | 0.02 | 76 | 953.47 | 3E-5 | 0.03 |
| 77 | 401.76 | 9.8E-4 | 0.39 | 77 | 959.86 | 0.00443 | 4.26 |
| 78 | 402.8 | 0 | 0 | 78 | 969.39 | 0 | 0 |
| 79 | 418.83 | 0.00637 | 2.67 | 79 | 974.25 | 0.00221 | 2.16 |
| 80 | 424.69 | 0.00561 | 2.38 | 80 | 1007.2 | 1E-5 | 0.01 |
| 81 | 428.48 | 0.00197 | 0.84 | 81 | 1020.33 | 0.02026 | 20.67 |
| 82 | 430.05 | 0.00303 | 1.3 | 82 | 1021.8 | 0.00274 | 2.8 |
| 83 | 432.16 | 0.0029 | 1.25 | 83 | 1022.35 | 5.4E-4 | 0.55 |
| 84 | 435.31 | 0.00635 | 2.77 | 84 | 1030.36 | 3E-5 | 0.04 |
| 85 | 437.95 | 0.00597 | 2.61 | 85 | 1031.53 | 2E-5 | 0.02 |
| 86 | 442.63 | 3E-5 | 0.01 | 86 | 1044.21 | 0 | 0 |
| 87 | 444.96 | 2E-5 | 0.01 | 87 | 1046.54 | 5E-5 | 0.05 |
| 88 | 450.43 | 0.00895 | 4.03 | 88 | 1048.67 | 3E-5 | 0.04 |
| 89 | 458.55 | 0.00258 | 1.18 | 89 | 1057.02 | 1.1E-4 | 0.12 |
| 90 | 463.59 | 0.00323 | 1.5 | 90 | 1062.19 | 0.03977 | 42.25 |
| 91 | 468.98 | 0.01203 | 5.64 | 91 | 1066.33 | 0.02768 | 29.52 |
| 92 | 474.48 | 3E-5 | 0.02 | 92 | 1072.52 | 0.00393 | 4.22 |

| | | | | | | | |
|---|---|---|---|---|---|---|---|
| 93 | 480.14 | 9E-5 | 0.04 | 93 | 1082.76 | 0.00555 | 6.01 |
| 94 | 484.36 | 1E-5 | 0 | 94 | 1090.66 | 0.00244 | 2.66 |
| 95 | 489.29 | 0.00102 | 0.5 | 95 | 1105.1 | 0.00114 | 1.26 |
| 96 | 495.44 | 3.3E-4 | 0.16 | 96 | 1106.68 | 3E-5 | 0.03 |
| 97 | 499.55 | 1.4E-4 | 0.07 | 97 | 1110.64 | 4E-5 | 0.05 |
| 98 | 502.46 | 2.7E-4 | 0.13 | 98 | 1128.17 | 0.00725 | 8.18 |
| 99 | 508.77 | 2E-5 | 0.01 | 99 | 1133.29 | 0.00174 | 1.98 |
| 100 | 537.51 | 8E-5 | 0.04 | 100 | 1136.38 | 0.00281 | 3.2 |
| 101 | 538.22 | 3.6E-4 | 0.19 | 101 | 1142.92 | 0.00159 | 1.81 |
| 102 | 538.77 | 3E-5 | 0.01 | 102 | 1150.62 | 0.00426 | 4.9 |
| 103 | 539.58 | 6E-5 | 0.03 | 103 | 1162.47 | 0.0129 | 15 |
| 104 | 544.95 | 3.3E-4 | 0.18 | 104 | 1170.56 | 4.8E-4 | 0.56 |
| 105 | 545.78 | 1E-5 | 0 | 105 | 1171.65 | 0.01354 | 15.87 |
| 106 | 545.86 | 2E-5 | 0.01 | 106 | 1171.88 | 0.00514 | 6.02 |
| 107 | 546.42 | 0 | 0 | 107 | 1177.29 | 0.00407 | 4.79 |
| 108 | 552.81 | 0.01336 | 7.39 | 108 | 1187.43 | 0.00355 | 4.22 |
| 109 | 562.51 | 1E-5 | 0 | 109 | 1203.1 | 0.0262 | 31.52 |
| 110 | 565.41 | 4.7E-4 | 0.27 | 110 | 1205.64 | 6.8E-4 | 0.83 |
| 111 | 573.82 | 8.7E-4 | 0.5 | 111 | 1219.08 | 0.00209 | 2.55 |
| 112 | 575.42 | 0.0038 | 2.19 | 112 | 1233.81 | 3.5E-4 | 0.43 |
| 113 | 577.84 | 0 | 0 | 113 | 1259.71 | 0.00228 | 2.87 |
| 114 | 578.3 | 5.4E-4 | 0.31 | 114 | 1275.09 | 0.00275 | 3.51 |
| 115 | 581.07 | 0.00304 | 1.77 | 115 | 1279.69 | 0.01291 | 16.52 |
| 116 | 587.03 | 9.5E-4 | 0.56 | 116 | 1291.45 | 9E-5 | 0.12 |
| 117 | 588.83 | 5.9E-4 | 0.35 | 117 | 1303.3 | 0.00222 | 2.9 |
| 118 | 590.11 | 1.3E-4 | 0.08 | 118 | 1312.49 | 0.00217 | 2.85 |
| 119 | 590.93 | 3E-4 | 0.18 | 119 | 1318.46 | 0.00294 | 3.87 |
| 120 | 596.62 | 2E-5 | 0.01 | 120 | 1325.37 | 0.00121 | 1.6 |
| 121 | 621.99 | 0.0174 | 10.82 | 121 | 1338.95 | 0.00142 | 1.91 |
| 122 | 631.16 | 0.00666 | 4.2 | 122 | 1352.28 | 0.00231 | 3.13 |
| 123 | 634.89 | 4E-5 | 0.02 | 123 | 1359.64 | 7.4E-4 | 1.01 |
| 124 | 637.17 | 0.00106 | 0.67 | 124 | 1360.63 | 0 | 0 |

| | | | | | | | |
|---|---|---|---|---|---|---|---|
| 125 | 639.07 | 6.5E-4 | 0.41 | 125 | 1362.99 | 1.1E-4 | 0.14 |
| 126 | 642.89 | 0.00375 | 2.41 | 126 | 1381.05 | 0.011 | 15.19 |
| 127 | 652.02 | 0.0026 | 1.69 | 127 | 1394.27 | 0.02336 | 32.56 |
| 128 | 656.26 | 0.01933 | 12.69 | 128 | 1398.69 | 0.0093 | 13.01 |
| 129 | 660.76 | 0.00147 | 0.97 | 129 | 1418.33 | 0.00637 | 9.03 |
| 130 | 663.14 | 3.6E-4 | 0.24 | 130 | 1442.63 | 0.00151 | 2.19 |
| 131 | 665 | 1E-5 | 0.01 | 131 | 1453.24 | 2.5E-4 | 0.37 |
| 132 | 668.21 | 0.00128 | 0.86 | 132 | 1465.77 | 6.8E-4 | 1 |
| 133 | 671.83 | 0.00831 | 5.58 | 133 | 1476.06 | 0.00155 | 2.28 |
| 134 | 676.36 | 0.00317 | 2.14 | 134 | 1477.26 | 5.6E-4 | 0.83 |
| 135 | 678.58 | 0.01764 | 11.97 | 135 | 1477.47 | 0.00104 | 1.54 |
| 136 | 684.59 | 0.00355 | 2.43 | 136 | 1484.2 | 0.00159 | 2.36 |
| 137 | 699.55 | 4.9E-4 | 0.35 | 137 | 1487.49 | 0.00397 | 5.91 |
| 138 | 719.27 | 0.00427 | 3.07 | 138 | 1490.59 | 0.00166 | 2.47 |
| 139 | 724.4 | 7.8E-4 | 0.56 | 139 | 1495.43 | 0.00273 | 4.09 |
| 140 | 732.42 | 1.5E-4 | 0.11 | 140 | 1500.74 | 0 | 0 |
| 141 | 734.58 | 1.2E-4 | 0.09 | 141 | 1518.04 | 0.00267 | 4.06 |
| 142 | 739.52 | 0.00428 | 3.16 | 142 | 1533.85 | 4.7E-4 | 0.72 |
| 143 | 763.08 | 1E-5 | 0.01 | 143 | 1541.3 | 1.6E-4 | 0.25 |
| 144 | 768.69 | 3.8E-4 | 0.3 | 144 | 1546.56 | 0.00597 | 9.23 |
| 145 | 774.68 | 3.2E-4 | 0.25 | 145 | 1568.41 | 7.8E-4 | 1.22 |
| 146 | 776.02 | 1E-5 | 0.01 | 146 | 1576.15 | 4E-5 | 0.07 |
| 147 | 776.74 | 0 | 0 | 147 | 1600.01 | 0 | 0 |
| 148 | 780.8 | 8.2E-4 | 0.64 | 148 | 1630.93 | 1.9E-4 | 0.3 |
| 149 | 781.99 | 0 | 0 | 149 | 1638.86 | 0.00482 | 7.89 |
| 150 | 789.35 | 0.00331 | 2.61 | 150 | 1649.33 | 7.2E-4 | 1.19 |
| 151 | 797.9 | 2E-5 | 0.02 | 151 | 1657.5 | 2E-5 | 0.04 |
| 152 | 799.13 | 8E-5 | 0.06 | 152 | 1679.18 | 0.0082 | 13.76 |
| 153 | 806.11 | 0 | 0 | 153 | 1684.15 | 0.00426 | 7.17 |
| 154 | 811.3 | 4.6E-4 | 0.37 | 154 | 1690.83 | 7E-5 | 0.12 |
| 155 | 813.9 | 7E-5 | 0.05 | 155 | 3046.91 | 8E-5 | 0.24 |
| 156 | 825.41 | 1E-5 | 0.01 | 156 | 3062.29 | 1E-5 | 0.02 |

| | | | | | | | |
|---|---|---|---|---|---|---|---|
| 157 | 860.41 | 0.00209 | 1.8 | 157 | 3158.44 | 3E-5 | 0.09 |
| 158 | 866.71 | 6E-4 | 0.52 | 158 | 3176.32 | 4E-5 | 0.14 |
| 159 | 869.12 | 5E-5 | 0.04 | 159 | 3182.81 | 3.4E-4 | 1.08 |
| 160 | 874.37 | 3E-5 | 0.03 | 160 | 3183.83 | 0 | 0 |
| 161 | 876.22 | 5E-5 | 0.04 | 161 | 3189.17 | 0 | 0.01 |
| 162 | 880.28 | 5.6E-4 | 0.49 | 162 | 3190.87 | 5E-5 | 0.16 |
| 163 | 881.99 | 3E-5 | 0.03 | 163 | 3192.86 | 0 | 0 |
| 164 | 886.19 | 6E-5 | 0.05 | 164 | 3198.16 | 1E-5 | 0.02 |
| 165 | 889.55 | 4.1E-4 | 0.37 | 165 | 3198.98 | 0.00671 | 21.47 |
| 166 | 891.78 | 2E-5 | 0.01 | 166 | 3199.66 | 0 | 0 |
| 167 | 892.76 | 0 | 0 | 167 | 3204.78 | 7E-5 | 0.22 |
| 168 | 893.52 | 2E-5 | 0.02 | 168 | 3206.66 | 1.1E-4 | 0.36 |
| 169 | 895.23 | 0 | 0 | 169 | 3207.12 | 0 | 0 |
| 170 | 898.95 | 9E-5 | 0.08 | 170 | 3212.4 | 1E-5 | 0.04 |
| 171 | 899.15 | 0 | 0 | 171 | 3212.42 | 0 | 0.01 |
| 172 | 904.13 | 3E-5 | 0.02 | 172 | 3216.76 | 0.00561 | 18.06 |
| 173 | 906.95 | 0.00111 | 1.01 | 173 | 3217.72 | 3E-5 | 0.08 |
| 174 | 912.13 | 3E-5 | 0.03 | 174 | 3224.15 | 2.4E-4 | 0.78 |
| 175 | 922.03 | 1E-4 | 0.09 | 175 | 3225.14 | 0 | 0.01 |
| 176 | 925.09 | 0 | 0 | 176 | 3229.63 | 0 | 0.01 |
| 177 | 925.76 | 0 | 0 | 177 | 3241.29 | 0 | 0 |
| 178 | 928.18 | 1E-5 | 0.01 | 178 | 3247.96 | 3E-5 | 0.1 |
| 179 | 930.26 | 1.4E-4 | 0.13 | 179 | 3248.84 | 2E-5 | 0.07 |
| 180 | 943.81 | 0.00226 | 2.13 | 180 | 3297.75 | 0 | 0 |
| 181 | 947.38 | 1E-5 | 0.01 | 181 | | | |
| 182 | 950.07 | 4.4E-4 | 0.42 | 182 | | | |
| 183 | 955.12 | 0 | 0 | 183 | | | |
| 184 | 955.54 | 0 | 0 | 184 | | | |
| 185 | 955.82 | 0 | 0 | 185 | | | |
| 186 | 959.15 | 4E-5 | 0.04 | 186 | | | |
| 187 | 959.93 | 5.4E-4 | 0.52 | 187 | | | |
| 188 | 965.4 | 0 | 0 | 188 | | | |

| | | | | |
|---|---|---|---|---|
| 189 | 966.94 | 8.6E-4 | 0.83 | 189 |
| 190 | 973.35 | 0 | 0 | 190 |
| 191 | 975.67 | 0 | 0 | 191 |
| 192 | 987.73 | 8E-5 | 0.08 | 192 |
| 193 | 988.26 | 0 | 0 | 193 |
| 194 | 989.71 | 8E-5 | 0.08 | 194 |
| 195 | 994.13 | 0 | 0 | 195 |
| 196 | 1013.6 | 5E-5 | 0.05 | 196 |
| 197 | 1016.6 | 0 | 0 | 197 |
| 198 | 1022.34 | 1E-5 | 0.01 | 198 |
| 199 | 1022.98 | 0 | 0 | 199 |
| 200 | 1024.6 | 0 | 0 | 200 |
| 201 | 1025.75 | 1.5E-4 | 0.16 | 201 |
| 202 | 1028.23 | 0 | 0 | 202 |
| 203 | 1029.31 | 0 | 0 | 203 |
| 204 | 1030.65 | 1E-5 | 0.01 | 204 |
| 205 | 1030.97 | 1E-5 | 0.01 | 205 |
| 206 | 1031.19 | 0 | 0 | 206 |
| 207 | 1034.18 | 3E-5 | 0.03 | 207 |
| 208 | 1034.81 | 2.8E-4 | 0.28 | 208 |
| 209 | 1035.28 | 0 | 0 | 209 |
| 210 | 1036.5 | 0 | 0 | 210 |
| 211 | 1039.63 | 0 | 0 | 211 |
| 212 | 1040.12 | 2.2E-4 | 0.23 | 212 |
| 213 | 1043.41 | 6.3E-4 | 0.66 | 213 |
| 214 | 1047.79 | 0 | 0 | 214 |
| 215 | 1049.79 | 0 | 0 | 215 |
| 216 | 1054.18 | 0 | 0 | 216 |
| 217 | 1054.38 | 1E-5 | 0.01 | 217 |
| 218 | 1054.95 | 8E-5 | 0.09 | 218 |
| 219 | 1055.1 | 0 | 0 | 219 |
| 220 | 1055.73 | 4E-5 | 0.04 | 220 |

| | | | | |
|---|---|---|---|---|
| 221 | 1056.33 | 0 | 0 | 221 |
| 222 | 1057.03 | 2E-5 | 0.02 | 222 |
| 223 | 1060.72 | 0 | 0 | 223 |
| 224 | 1061.19 | 0 | 0 | 224 |
| 225 | 1061.35 | 0 | 0 | 225 |
| 226 | 1064.95 | 1.9E-4 | 0.2 | 226 |
| 227 | 1065.95 | 3E-5 | 0.03 | 227 |
| 228 | 1066.8 | 4E-4 | 0.42 | 228 |
| 229 | 1067.76 | 0 | 0 | 229 |
| 230 | 1071.76 | 0.00153 | 1.64 | 230 |
| 231 | 1077.43 | 0.0101 | 10.88 | 231 |
| 232 | 1088.1 | 0 | 0 | 232 |
| 233 | 1103.78 | 1.3E-4 | 0.14 | 233 |
| 234 | 1112.12 | 0 | 0 | 234 |
| 235 | 1120.03 | 0 | 0 | 235 |
| 236 | 1127.66 | 4E-5 | 0.04 | 236 |
| 237 | 1129.18 | 0.00286 | 3.23 | 237 |
| 238 | 1130.35 | 5.9E-4 | 0.67 | 238 |
| 239 | 1132.07 | 0.00153 | 1.73 | 239 |
| 240 | 1138.06 | 1E-5 | 0.02 | 240 |
| 241 | 1153.7 | 0.01754 | 20.24 | 241 |
| 242 | 1161.84 | 0.00577 | 6.7 | 242 |
| 243 | 1172.67 | 1E-5 | 0.01 | 243 |
| 244 | 1174.19 | 8E-5 | 0.09 | 244 |
| 245 | 1178.35 | 5E-5 | 0.06 | 245 |
| 246 | 1180.06 | 2.5E-4 | 0.29 | 246 |
| 247 | 1182.61 | 6E-4 | 0.71 | 247 |
| 248 | 1183.29 | 0.00106 | 1.25 | 248 |
| 249 | 1185.78 | 1E-4 | 0.12 | 249 |
| 250 | 1192.41 | 0 | 0 | 250 |
| 251 | 1195.56 | 6.6E-4 | 0.79 | 251 |
| 252 | 1202.82 | 0.01423 | 17.11 | 252 |

| | | | | |
|---|---|---|---|---|
| 253 | 1203.05 | 0.00112 | 1.35 | 253 |
| 254 | 1208.91 | 3.3E-4 | 0.4 | 254 |
| 255 | 1210.57 | 2.2E-4 | 0.26 | 255 |
| 256 | 1218.25 | 0.00172 | 2.09 | 256 |
| 257 | 1239.07 | 8.1E-4 | 1 | 257 |
| 258 | 1243.73 | 0.00253 | 3.15 | 258 |
| 259 | 1247.69 | 0.00286 | 3.56 | 259 |
| 260 | 1255.17 | 9E-5 | 0.11 | 260 |
| 261 | 1260.42 | 3E-5 | 0.03 | 261 |
| 262 | 1261.32 | 1E-5 | 0.01 | 262 |
| 263 | 1261.93 | 0 | 0 | 263 |
| 264 | 1263.14 | 0 | 0 | 264 |
| 265 | 1279.8 | 7.4E-4 | 0.94 | 265 |
| 266 | 1285.77 | 0.00185 | 2.38 | 266 |
| 267 | 1290.28 | 0.0013 | 1.68 | 267 |
| 268 | 1294.82 | 0.00136 | 1.77 | 268 |
| 269 | 1297.38 | 0.00118 | 1.53 | 269 |
| 270 | 1301.67 | 2.9E-4 | 0.37 | 270 |
| 271 | 1307.75 | 0.03401 | 44.48 | 271 |
| 272 | 1314.26 | 8E-5 | 0.11 | 272 |
| 273 | 1315.48 | 3.1E-4 | 0.41 | 273 |
| 274 | 1318.43 | 0.00392 | 5.17 | 274 |
| 275 | 1321.31 | 0 | 0 | 275 |
| 276 | 1321.76 | 1E-5 | 0.01 | 276 |
| 277 | 1322.19 | 0 | 0 | 277 |
| 278 | 1323.12 | 0 | 0 | 278 |
| 279 | 1328.14 | 3E-4 | 0.4 | 279 |
| 280 | 1333.06 | 0 | 0 | 280 |
| 281 | 1334.75 | 1.1E-4 | 0.15 | 281 |
| 282 | 1337.78 | 5E-5 | 0.06 | 282 |
| 283 | 1338.96 | 1E-4 | 0.13 | 283 |
| 284 | 1340.73 | 1.7E-4 | 0.22 | 284 |

| | | | | |
|---|---|---|---|---|
| 285 | 1342.06 | 2.9E-4 | 0.39 | 285 |
| 286 | 1351.2 | 4.2E-4 | 0.57 | 286 |
| 287 | 1360.16 | 0.00155 | 2.1 | 287 |
| 288 | 1363.86 | 3.4E-4 | 0.47 | 288 |
| 289 | 1366.52 | 5.2E-4 | 0.7 | 289 |
| 290 | 1370.8 | 0.00197 | 2.7 | 290 |
| 291 | 1380.26 | 0.00254 | 3.51 | 291 |
| 292 | 1384.19 | 5.2E-4 | 0.72 | 292 |
| 293 | 1389.53 | 1.7E-4 | 0.24 | 293 |
| 294 | 1401.84 | 7E-5 | 0.09 | 294 |
| 295 | 1402.86 | 0.00697 | 9.78 | 295 |
| 296 | 1402.9 | 1.8E-4 | 0.25 | 296 |
| 297 | 1404 | 0.0053 | 7.44 | 297 |
| 298 | 1404.39 | 1E-5 | 0.02 | 298 |
| 299 | 1404.6 | 0.00878 | 12.34 | 299 |
| 300 | 1405.97 | 0 | 0 | 300 |
| 301 | 1414.61 | 0.00177 | 2.5 | 301 |
| 302 | 1418.75 | 0 | 0 | 302 |
| 303 | 1419.84 | 1E-5 | 0.02 | 303 |
| 304 | 1420.92 | 0 | 0 | 304 |
| 305 | 1428.3 | 3.9E-4 | 0.55 | 305 |
| 306 | 1445.88 | 0 | 0 | 306 |
| 307 | 1447.18 | 0 | 0 | 307 |
| 308 | 1448.65 | 0 | 0 | 308 |
| 309 | 1457.5 | 0 | 0 | 309 |
| 310 | 1458.48 | 1E-5 | 0.01 | 310 |
| 311 | 1461.4 | 1E-5 | 0.01 | 311 |
| 312 | 1461.89 | 4.3E-4 | 0.63 | 312 |
| 313 | 1462.29 | 0 | 0 | 313 |
| 314 | 1462.46 | 1.7E-4 | 0.24 | 314 |
| 315 | 1463.65 | 1E-5 | 0.01 | 315 |
| 316 | 1463.84 | 2E-5 | 0.03 | 316 |

| | | | | |
|---|---|---|---|---|
| 317 | 1464.39 | 1.9E-4 | 0.27 | 317 |
| 318 | 1464.49 | 0 | 0 | 318 |
| 319 | 1465 | 0 | 0 | 319 |
| 320 | 1465.5 | 0 | 0 | 320 |
| 321 | 1467.52 | 0 | 0 | 321 |
| 322 | 1467.92 | 0 | 0.01 | 322 |
| 323 | 1467.98 | 2E-5 | 0.03 | 323 |
| 324 | 1468.4 | 2E-5 | 0.03 | 324 |
| 325 | 1469.56 | 0 | 0 | 325 |
| 326 | 1471.79 | 0 | 0 | 326 |
| 327 | 1482.6 | 5.3E-4 | 0.78 | 327 |
| 328 | 1488.69 | 0.00158 | 2.35 | 328 |
| 329 | 1491.55 | 6E-5 | 0.09 | 329 |
| 330 | 1498.75 | 0 | 0 | 330 |
| 331 | 1499.58 | 0 | 0 | 331 |
| 332 | 1499.81 | 0 | 0 | 332 |
| 333 | 1501.33 | 1.6E-4 | 0.24 | 333 |
| 334 | 1505.96 | 4.3E-4 | 0.65 | 334 |
| 335 | 1509.14 | 0.00401 | 6.05 | 335 |
| 336 | 1519.01 | 0.01403 | 21.32 | 336 |
| 337 | 1529.81 | 3E-5 | 0.05 | 337 |
| 338 | 1531.52 | 1.7E-4 | 0.26 | 338 |
| 339 | 1534.55 | 0.00219 | 3.36 | 339 |

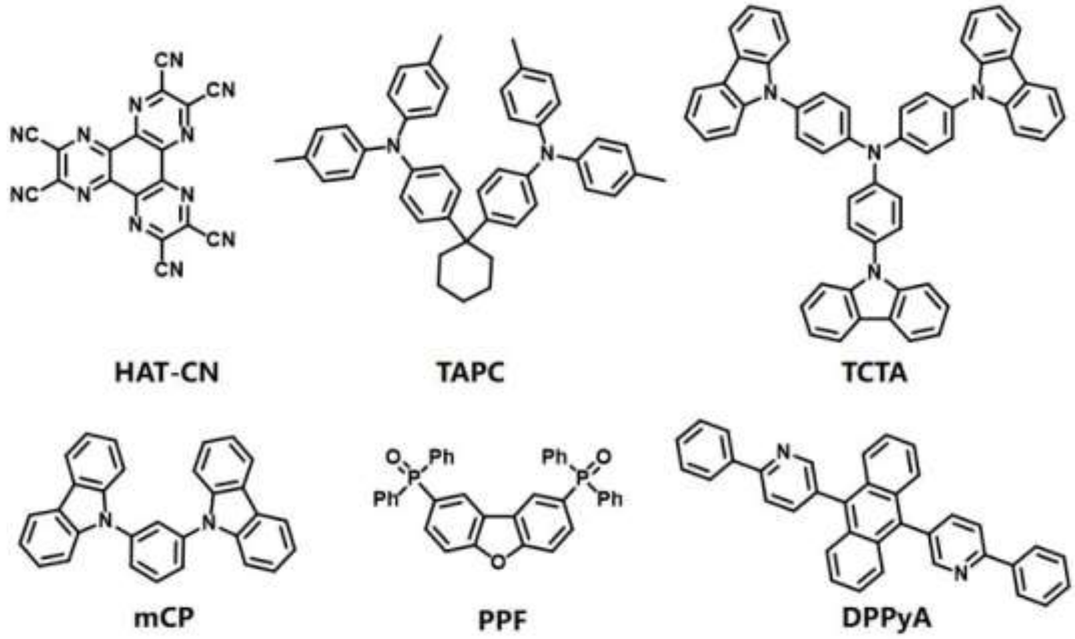


**Figure. S16.** Molecular structures of the materials used in devices.

**Table S4.** Summary of the EL data of BN-CP based OLED.

| Device | $\lambda_{EL}$[a] [nm] | FWHM[a] [nm] | $V_{turn-on}$[b] [V] | EQE[c] [%] | CE[c] [cd/A] | CIE[d] (x,y) |
|---|---|---|---|---|---|---|
| 1.5 wt% | 433 | 32 | 3.0 | 23.3/17.3/8.5 | 23.2/18.9/9.7 | 0.16,0.04 |

[a]Maximum electroluminescence wavelength. [†]Full width at half maximum of electroluminescence. [b]Turn-on voltage when brightness is 1 cd $m^{-2}$. [c]Maximum efficiency/ efficiency at 100 cd $m^{-2}$/efficiency at 1000 cd $m^{-2}$. [d]Recorded at 10 mA $cm^{-1}$.

**Table S5.** Summary of reported blue TADF-OLED with CIEy ≤ 0.05.

| Emitter | Host | $\lambda_{EL}$ / FWHM [nm] | $EQE_{max}$ [%] | CIE (x, y) | Ref. |
|---|---|---|---|---|---|
| BOC-PSi | DPEPO | 433 / 53 | 19.6 | 0.154, 0.049 | [1] |
| DMACN-B | DPEPO | 444 / 44 | 10.0 | 0.151, 0.045 | [2] |
| TB-tPCz | mCP | 428 / 42 | 15.8 | 0.16, 0.05 | [3] |
| TDBA-PAS | DPEPO | 435 / 50 | 22.35 | 0.155, 0.042 | [4] |
| f-DOABNA | mCP | 445 / 24 | 19.5 | 0.150, 0.041 | [5] |
| BIC-mCz | PPF | 432 / 42 | 19.4 | 0.16, 0.05 | [6] |
| m-DBIC | PPF | 431 / 30 | 13.5 | 0.16, 0.05 | |
| CZCO | mCP | 432 / 35 | 15.6 | 0.154, 0.047 | [7] |
| BOBO-Z | mCBP | 445 / 18 | 13.6 | 0.15, 0.04 | [8] |
| B-O-dpa | DPEPO | 443 / 32 | 16.3 | 0.15, 0.05 | [9] |
| DB | DOBNA-OAr | 443 / 26 | 23.4 | 0.154, 0.048 | [10] |
| DB-O | DOBNA-OAr | 445 / 24 | 27.5 | 0.150, 0.041 | |
| DB-S | DOBNA-OAr | 447 / 24 | 29.3 | 0.148, 0.047 | |
| 5Cz-BO | TSPO1 | 416 / 36 | 22.8 | 0.163, 0.046 | [11] |
| Py-BN | DOBNA-OAr | 444 / 21 | 15.8 | 0.153, 0.045 | [12] |
| Pm-BN | DOBNA-OAr | 415 / 24 | 5.8 | 0.161, 0.045 | |
| BN-CP | PPF | 432 / 32 | 23.3 | 0.16, 0.04 | **This work** |

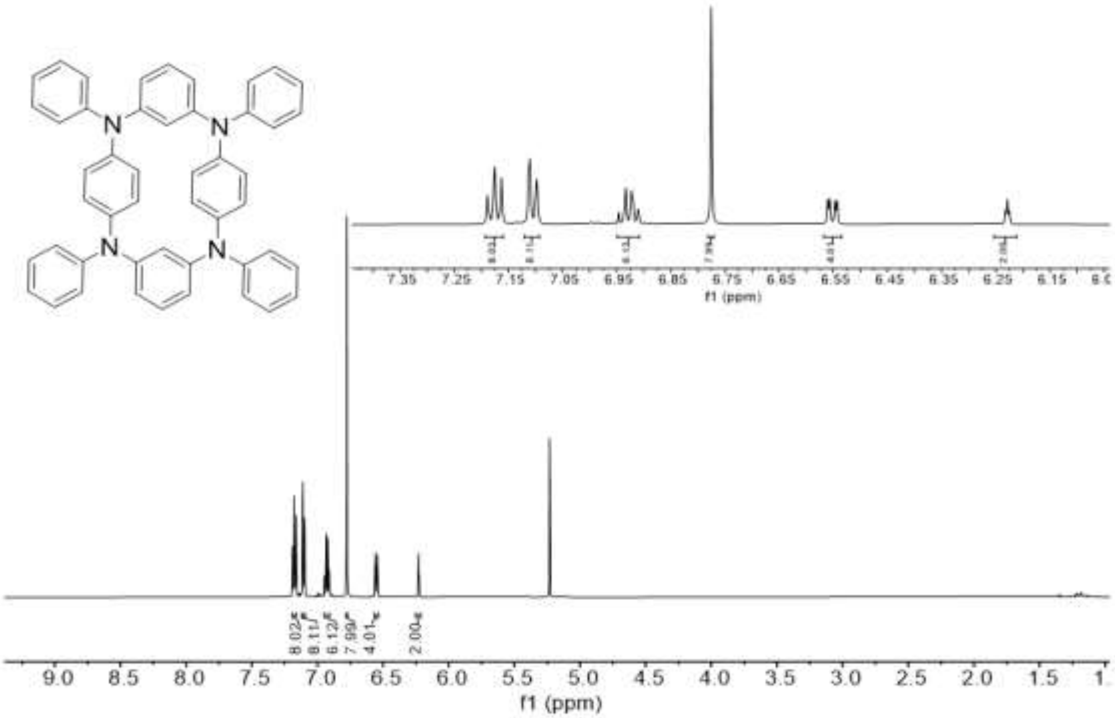

**Figure. S17.** $^{1}H$ NMR spectrum of intermediate **1** in $CD_2Cl_2$.

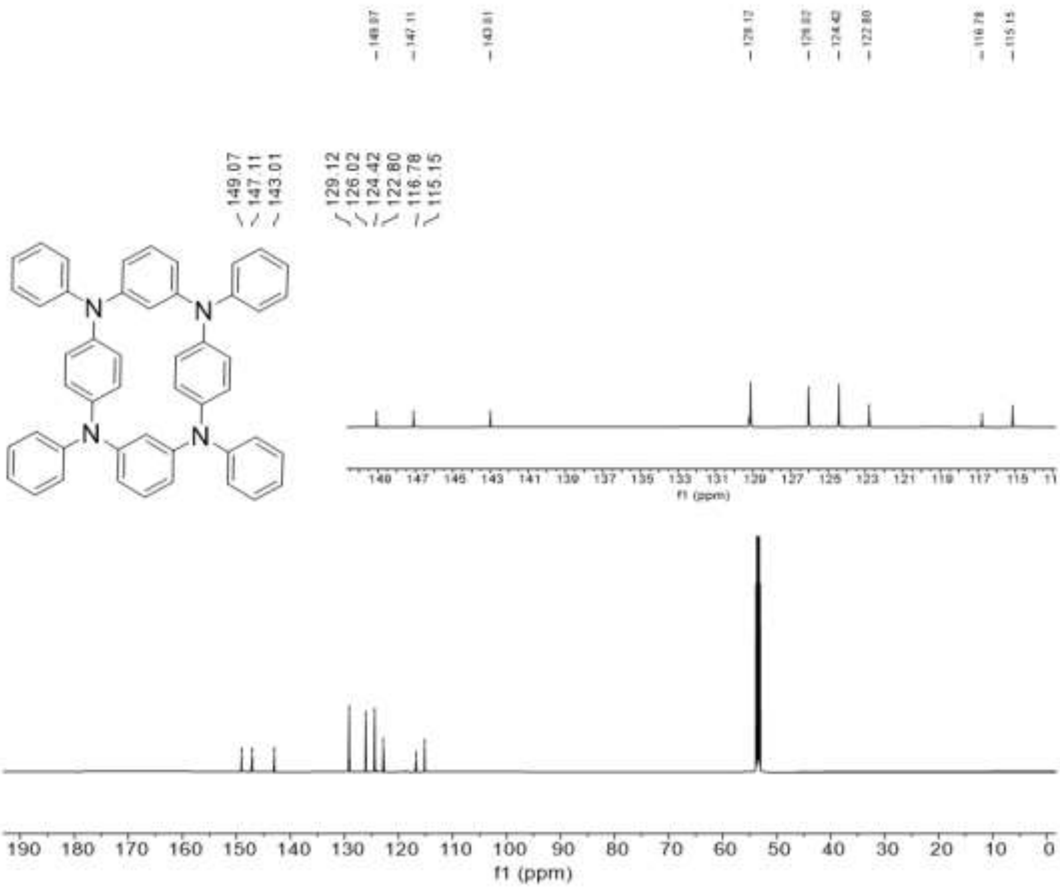


**Figure. S18.** $^{13}$C NMR spectrum of intermediate **1** in $CD_2Cl_2$.

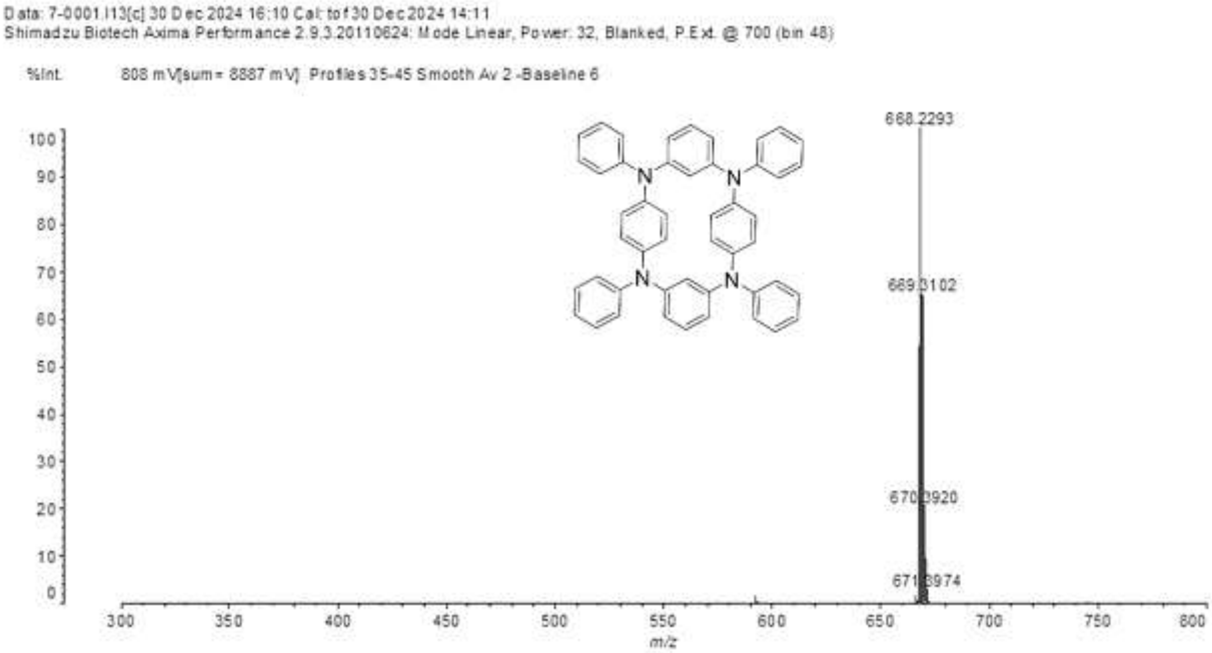


**Figure. S19.** High-resolution mass spectra (HRMS) of intermediate **1**.

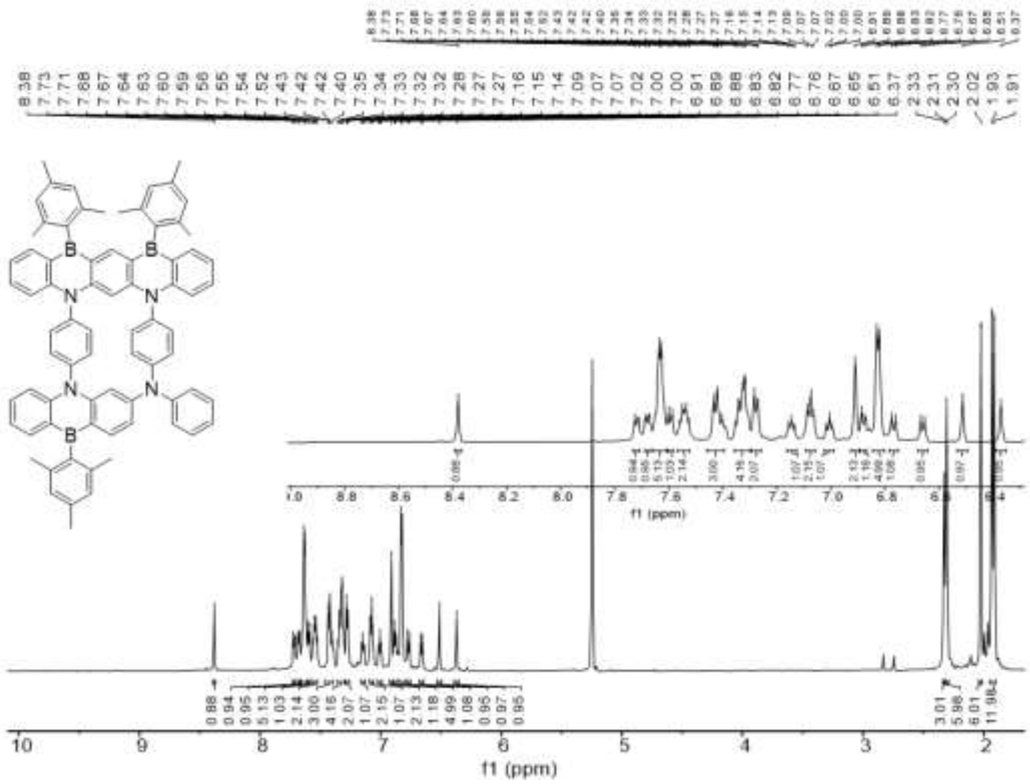

**Figure. S20.** $^{1}H$ NMR spectrum of BN-CP in $CD_2Cl_2$.

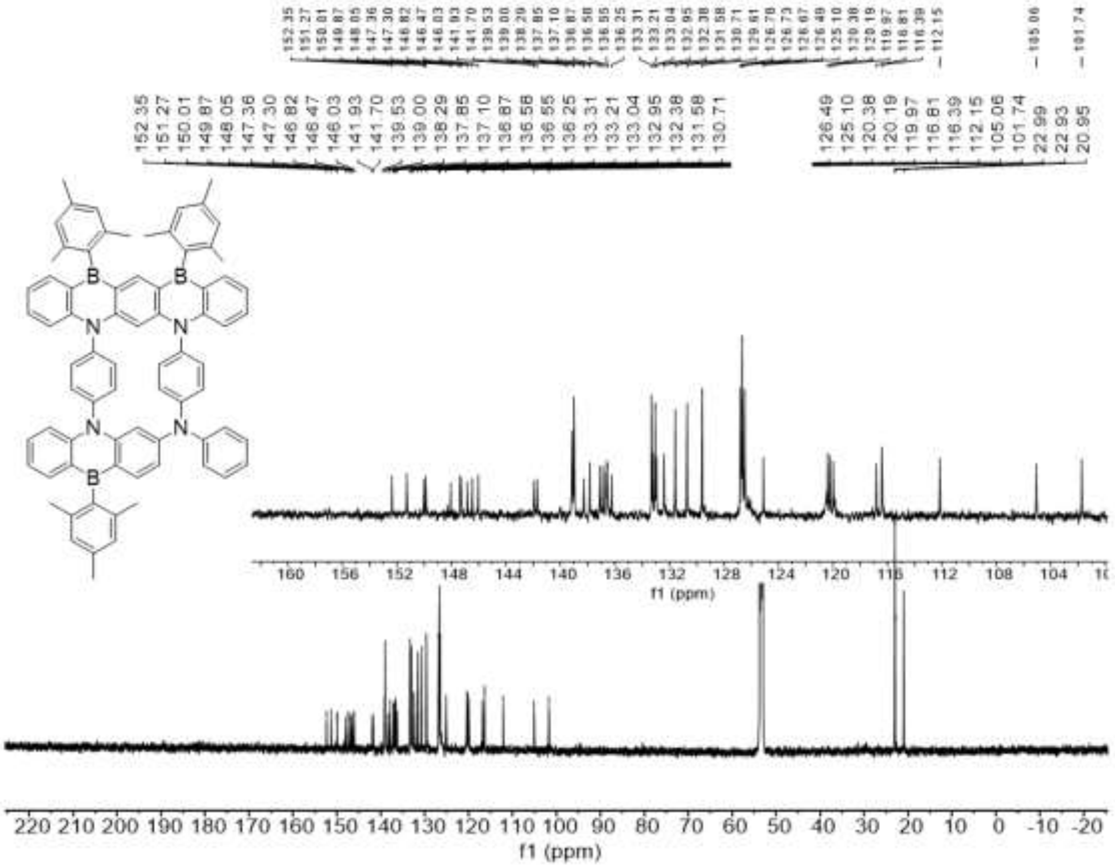

**Figure. S21.** $^{13}$C NMR spectrum of BN-CP in $CD_2Cl_2$.

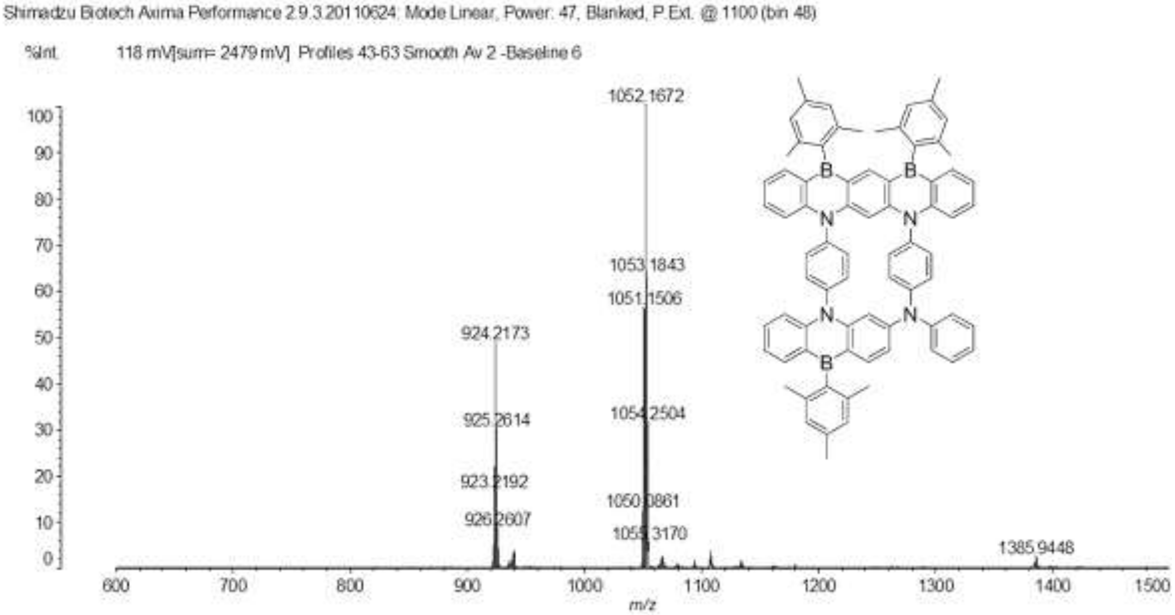


**Figure. S22.** High-resolution mass spectra (HRMS) of BN-CP.

**Reference**


1. Hua T, Cao X, Miao J, et al., Deep-blue organic light-emitting diodes for ultrahigh-definition displays. *Nat Photonics* 2024; **18**: 1161-1169.

2. Khan A, Tang X, Zhong C, et al., Intramolecular-Locked High Efficiency Ultrapure Violet-Blue (CIE-y <0.046) Thermally Activated Delayed Fluorescence Emitters Exhibiting Amplified Spontaneous Emission. *Adv Funct Mater* 2021; **31**: 2009488.

3. Kim H J, Kang H, Jeong J-E, et al., Ultra-Deep-Blue Aggregation-Induced Delayed Fluorescence Emitters: Achieving Nearly 16% EQE in Solution-Processed Nondoped and Doped OLEDs with CIE < 0.1. *Adv Funct Mater* 2021; **31**: 2102588.

4. Tan H-J, Yang G-X, Deng Y-L, et al., Deep-Blue OLEDs with Rec.2020 Blue Gamut Compliance and EQE Over 22% Achieved by Conformation Engineering. *Adv Mater* 2022; **34**: 2200537.

5. Weerasinghe R W, Madayanad Suresh S, Hall D, et al., A Boron, Nitrogen, and Oxygen Doped π-Extended Helical Pure Blue Multiresonant Thermally Activated Delayed Fluorescent Emitter for Organic Light Emitting Diodes That Shows Fast kRISC Without the Use of Heavy Atoms. *Adv Mater* 2024; **36**: 2402289.

6. Wang X, Zhang Y, Dai H, et al., Mesityl-Functionalized Multi-Resonance Organoboron Delayed Fluorescent Frameworks with Wide-Range Color Tunability for Narrowband OLEDs. *Angew Chem Int Ed* 2022; **61**: e202206916.

7. Cao C, Tan J-H, Zhu Z-L, et al., Intramolecular Cyclization: A Convenient Strategy to Realize Efficient BT.2020 Blue Multi-Resonance Emitter for Organic Light-Emitting Diodes. *Angew Chem Int Ed* 2023; **62**: e202215226.

8. Park I S, Yang M, Shibata H, et al., Achieving Ultimate Narrowband and Ultrapure Blue Organic Light-Emitting Diodes Based on Polycyclo-Heteraborin Multi-Resonance Delayed-Fluorescence Emitters. *Adv Mater* 2022; **34**: 2107951.

9. Park J, Lim J, Lee J H, et al., Asymmetric Blue Multiresonance TADF Emitters with a Narrow Emission Band. *ACS Appl Mater Interfaces* 2021; **13**: 45798-45805.

10. Ye Z, Wu H, Xu Y, et al., Deep-Blue Narrowband Hetero[6]helicenes Showing Circularly Polarized Thermally Activated Delayed Fluorescence Toward High-Performance OLEDs. *Adv Mater* 2024; **36**: 2308314.

11. An R-Z, Sun Y, Chen H-Y, et al., Excited-State Engineering Enables Efficient Deep-Blue Light-Emitting Diodes Exhibiting BT.2020 Color Gamut. *Adv Mater* 2024; **36**: 2313602.

12. Cai X, Pan Y, Li C, *et al.*, Nitrogen-Embedding Strategy for Short-Range Charge Transfer Excited States and Efficient Narrowband Deep-Blue Organic Light Emitting Diodes. *Angew Chem Int Ed* 2024; **63**: e202408522.
